# DeepChIA-PET: Accurately predicting ChIA-PET from Hi-C and ChIP-seq with deep dilated networks

**DOI:** 10.1371/journal.pcbi.1011307

**Published:** 2023-07-13

**Authors:** Tong Liu, Zheng Wang

**Affiliations:** Department of Computer Science, University of Miami, Coral Gables, Florida, United States of America; La Jolla Institute for Allergy and Immunology, UNITED STATES

## Abstract

Chromatin interaction analysis by paired-end tag sequencing (ChIA-PET) can capture genome-wide chromatin interactions mediated by a specific DNA-associated protein. The ChIA-PET experiments have been applied to explore the key roles of different protein factors in chromatin folding and transcription regulation. However, compared with widely available Hi-C and ChIP-seq data, there are not many ChIA-PET datasets available in the literature. A computational method for accurately predicting ChIA-PET interactions from Hi-C and ChIP-seq data is needed that can save the efforts of performing wet-lab experiments. Here we present DeepChIA-PET, a supervised deep learning approach that can accurately predict ChIA-PET interactions by learning the latent relationships between ChIA-PET and two widely used data types: Hi-C and ChIP-seq. We trained our deep models with CTCF-mediated ChIA-PET of GM12878 as ground truth, and the deep network contains 40 dilated residual convolutional blocks. We first showed that DeepChIA-PET with only Hi-C as input significantly outperforms Peakachu, another computational method for predicting ChIA-PET from Hi-C but using random forests. We next proved that adding ChIP-seq as one extra input does improve the classification performance of DeepChIA-PET, but Hi-C plays a more prominent role in DeepChIA-PET than ChIP-seq. Our evaluation results indicate that our learned models can accurately predict not only CTCF-mediated ChIA-ET in GM12878 and HeLa but also non-CTCF ChIA-PET interactions, including RNA polymerase II (RNAPII) ChIA-PET of GM12878, RAD21 ChIA-PET of GM12878, and RAD21 ChIA-PET of K562. In total, DeepChIA-PET is an accurate tool for predicting the ChIA-PET interactions mediated by various chromatin-associated proteins from different cell types.

## Author summary

Various techniques have been widely used to model and investigate three-dimensional (3D) genomes, such as Hi-C, ChIA-PET, and HiChIP. Unlike Hi-C, ChIA-PET can capture genome-wide chromosomal contacts mediated by a predefined DNA-associated protein. Compared with an adequate resource of Hi-C data captured from different cell lines for various species, only a limited number of ChIA-PET data sets are publicly available. Here we developed a deep learning method using Hi-C and ChIP-seq data to predict ChIA-PET directly. Our evaluation results demonstrate that our learned models can successfully predict ChIA-PETs mediated by a different DNA-associated protein for a separate cell line. Our method is an effective alternative to obtaining ChIA-PET contacts from a wet lab.

## Introduction

Chromatin interaction analysis by paired-end tag sequencing (ChIA-PET) [1] is a technique that processes chromatin immunoprecipitation (ChIP)-enriched chromatin complexes by linker ligation, proximity ligation, and high-throughput sequencing to identify significant long-range chromatin interactions at the whole genome. Compared with Hi-C [2], the main advantage of ChIA-PET is that the interactions it captured are located at the binding sites of one specific DNA-associated protein of interest, such as the insulator binding protein CTCF [3], the RAD21 subunit of the cohesin complex [4,5], and RNA polymerase II (RNAPII) [6].

The CTCF-mediated and RAD21-mediated ChIA-PET experiments can be applied to investigate their key roles in chromatin folding and the establishment of topologically associating domains [7]. The RNAPII-mediated ChIA-PET interactions are an excellent source of studying transcription regulation. The genome-wide chromatin interactions captured by the Hi-C technique and its variants [8] can be thought of as a pool of ChIA-PET interactions including all chromatin-associated proteins, from which we can hardly identify interactions that are only related to one specific protein factor. Therefore, ChIA-PET is more applicable than Hi-C if exploring the potential functions of different DNA-associated proteins in the three-dimensional (3D) genome is of interest.

The chromatin immunoprecipitation followed by high-throughput sequencing (ChIP-seq) is a widely used method for analyzing interactions between DNA and chromatin-associated proteins, which can provide the binding sites of protein factors. Technically, we can think of ChIA-PET as a hybrid of Hi-C and ChIP-seq. However, there are not enough experimental ChIA-PET datasets publicly available in the literature: based on the NCBI GEO datasets, there are only 747 ChIA-PET experimental sets compared with 6934 Hi-C and 85,912 ChIP-seq sets as of May 2023. Therefore, computationally predicting ChIA-PET interactions from Hi-C and ChIP-seq is a promising way to enrich ChIA-PET datasets.

There are some computational methods in the literature designed for processing ChIA-PET data and directly detecting peaks, such as Mango [9], cLoops [10], and ChIAMM [11]. There are also some computational methods for predicting CTCF-mediated chromatin loops from DNA sequence-based features, such as CLNN-loop [12] and CTCF-MP [13], from various genomic and epigenomic features, such as Lollipop [14], and from CTCF and Hi-C data, such as LOOPbit [15]. However, none of these computational methods are targeted for learning and predicting ChIA-PET interactions.

Loop-Extrusion-Model [16] used a simple mathematical model of CTCF-mediated loop formation for predicting CTCF ChIA-PET loops. The limitations of this method are that the authors did not blindly test on non-CTCF ChIA-PET and widely available Hi-C data are not used as reference input. Peakachu [17] overcame the second limitation by directly using Hi-C to predict ChIA-PET interactions. However, Peakachu has the following disadvantages: (1) using only Hi-C data as input may result in the learned models being ChIP-specific. This is because bulk Hi-C data is not protein specific. Therefore, the machine learning model is trained to map non-protein-specific Hi-C data to protein-specific ChIA-PET interactions. Next time when the same machine learning model is used to predict the ChIA-PET interactions formed by a different type of protein, the input will be the same, which may cause the problem that the machine learning model may only be adapted to or work well for the type of ChIA-PET data used for training. In comparison, adding the ChIP-seq data in input as we have done in this research solves this problem to some extent. (2) the receptive field of machine learning was restricted to a 11×11 window for representing the Hi-C features of the center pixel; (3) the number of negative pixels for training was set equal to the number of positive pixels, resulting in millions of negative pixels never seen by the machine learning method; and (4) the strategy of one positive pixel as an independent training sample sacrifices the valuable information that one anchor may involve in multiple long-range ChIA-PET interactions.

In this paper, we present DeepChIA-PET, a deep-learning method for accurately predicting ChIA-PET interactions based on Hi-C and ChIP-seq data. DeepChIA-PET applied a deep dilated, residual convolutional network to learn the mapping from Hi-C and ChIP-seq to ChIA-PET at 10-kb and 5-kb resolution, which makes receptive fields larger enough for capturing long-range interactions and lets the deep learning method see all positive and negative pixels. Our evaluation results indicate that DeepChIA-PET significantly outperforms Peakachu, and our models trained with CTCF ChIA-PET can be accurately applied to predict non-CTCF ChIA-PET.

## Materials and methods

### Data processing for Hi-C, ChIP-seq, and ChIA-PET

We downloaded KR-normalized Hi-C data for five different cell types (GM12878, HeLa, K562, HMEC, and NHEK) at the resolutions of 10 kb and 5 kb using Juicer [18] (S1 Table), which are also available on Gene Expression Omnibus (GEO) under accession number GSE63525 [8]. Hi-C peaks for GM12878, HMEC, and NHEK were detected with HiCCUPS [8] at 10-kb resolution. TAD annotations of GM12878 at 10-kb resolution were downloaded from TADKB [19], which used the directionality index (DI) [7] to call TADs.

The ChIP-seq data for three different DNA-associated proteins (CTCF, RNAPII, and RAD21) were downloaded from UCSC website (S2 Table). The average value for each 10-kb or 5-kb bin was calculated by pyBigWig (https://github.com/deeptools/pyBigWig).

We used various ChIA-PET datasets in this study (S3 Table). The CTCF-mediated and RNAPII-mediated ChIA-PET interactions in GM12878 and HeLa were downloaded from GEO under accession number GSE72816. The RAD21-mediated ChIA-PET interactions for GM12878 and K562 were obtained from S1 Table in [4]. Since the anchor regions of ChIA-PET interactions are not binned at a given resolution, we assign one (positive) to pixels in a 2D ChIA-PET contact matrix at 10-kb resolution if the pixels overlap with two anchor regions of any known ChIA-PET interactions (referred to as positives), and zero otherwise. Since the lengths of almost all anchors are less than 10,000 bp (S1A Fig), the number of positive pixels is usually somewhat fewer than the number of raw ChIA-PET interactions.

We tested two resolutions (10 kb and 5 kb) for all three data types (i.e., Hi-C, ChIP-seq, and ChIA-PET) in this study. At the resolution of 10 kb, the Hi-C samples for training, validation, and blind test were extracted along the diagonal of a 2D contact matrix with a sliding window of 250×250 and a step of 50 bins. The ChIP-seq samples were obtained along each chromosome with a sliding window of 250 bins and a step of 50 bins. At the resolution of 5 kb, the window and step sizes for both Hi-C and ChIP-seq were changed to 500 and 100, respectively. Since the genomic distances of almost all ChIA-PET interactions are less than 2 Mb (S1B Fig), the genomic distance we covered by 250 bins (≤ 2.5 Mb) is long enough.

For each Hi-C contact matrix, we first rescaled the contacts by log transformation of log_2_(*x*+1) and then further rescaled all contacts to the range [0, 1] by min-max normalization. We calculated the mean and standard deviation (SD) of all Hi-C samples to perform final z-score normalization for each rescaled Hi-C matrix. For normalizing ChIP-seq vectors, we first did min-max normalization and then did z-score normalization as in normalizing Hi-C. The means and SDs for normalizing Hi-C and ChIP-seq were obtained from Hi-C and CTCF ChIP-seq of GM12878 and used on all the other testing datasets.

### Training, validation, and blind testing

Our deep models were trained with CTCF-mediated ChIA-PET of GM12878 as ground truth. The input data for training are the Hi-C and CTCF ChIP-seq data of GM12878. For blind testing on each chromosome from 1 up to X and to avoid data leakage, we trained 23 separate models. Specifically, excluding the current testing chromosome, the validation data were extracted from the largest remaining chromosome, and the rest chromosomes were used for generating the training data. For example, the model tested on chromosome 1 was trained on chromosomes 3 to X and validated on chromosome 2 (the largest chromosome in the remaining chromosomes). The 23 learned models were also used for testing/predicting both CTCF and non-CTCF ChIA-PET, specifically CTCF ChIA-PET of four cell types (GM12878, HeLa, HMEC, and NHEK), RANPII ChIA-PET of GM12878 and HeLa, and RAD21 ChIA-PET of GM12878 and K562.

When a user executes the standalone version of DeepChIA-PET, if the predictions are made on a human chromosome, the model that is tested on that chromosome is used to make predictions. For example, if a user wants to make predictions on human chromosome 1, then the model trained on chromosomes 3 to X and validated on chromosome 2 will be used. If the user makes predictions for any non-human chromosome, the model trained on human chromosomes 3 to X and validated on human chromosome 2 will be used. Our evaluation results in the later sections will show that the predictions from different models are accurate and stable.

### DeepChIA-PET architecture

**Fig 1:**
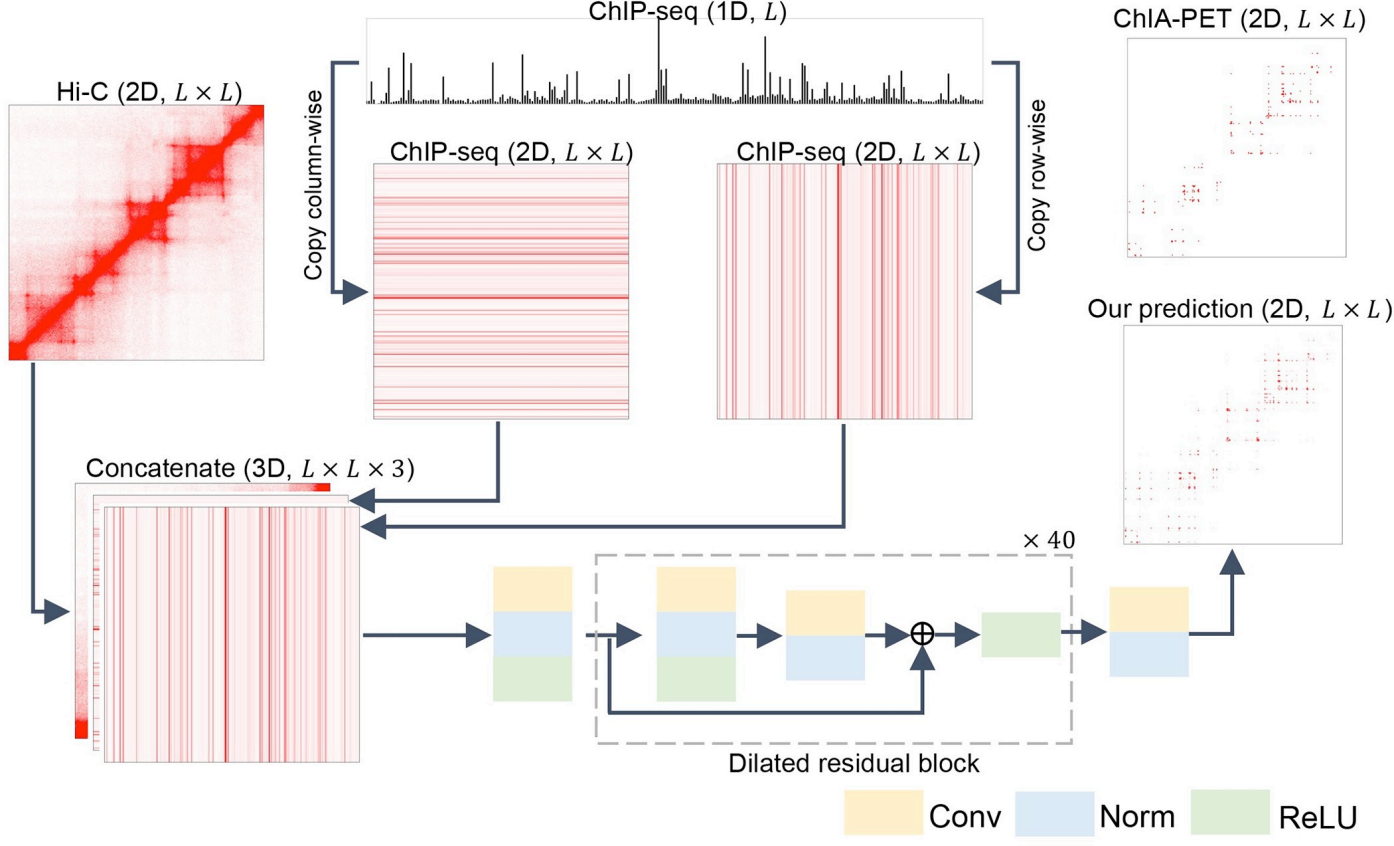
Framework of DeepChIA-PET. The 1D sequential ChIP-seq data are converted into two 2D pairwise matrices, which are further concatenated with Hi-C. The dashed box contains a typical residual block.

We illustrated the pipeline of DeepChIA-PET in Fig 1. The original inputs are 2D Hi-C contact matrices and one-dimensional (1D) ChIP-seq vectors. To feed 1D ChIP-seq into our 2D convolutional network, we first converted the 1D ChIP-seq data into 2D by copying the ChIP-seq vector column- and row-wise, resulting in two 2D ChIP-seq matrices. We then concatenated the Hi-C and ChIP-seq matrices and obtained a 3D tensor as input.

The final deep network we used is inspired by two deep networks for the prediction of protein contact maps [20,21] and contains three main parts. The first part consists of a 2D convolutional layer (Conv1 with 1×1 kernel size) for enhancing the hidden dimension from three to 128, a batch normalization layer, and a ReLU [22]. The second part consists of 40 typical residual blocks [23] for learning the latent relationship between ChIA-PET and our inputs. Each block contains two dilated 2D convolutional layers (Conv2 with 3×3 kernel size) [24]. We set different dilation values for the two Conv2 layers in each block for capturing multi-scale, long-range ChIA-PET interactions. The last part consists of a 2D convolutional layer (Conv3 with 1×1 kernel size) and a batch normalization layer for reducing the output channel from 128 to one.

Since we may predict one pixel more than one time, the predicted score for the pixel is the average value of all its predicted scores. Moreover, since ChIA-PET interaction matrices should be symmetric, we further averaged the predicted values in the upper and lower triangular matrices to get the final predicted scores. After obtaining the final predicted score for each pixel, we sort all predicted pixels by their predicted probabilities in descending order.

### Implementation details

We implemented our network in PyTorch [25]. The optimizer we used was Adam [26] with a weight decay of 1e-04. We tested five different batch sizes (4, 8, 16, 32, and 64) and used two strategies for setting the learning rate. The first one is to use a fixed value (0.01, 0.001, and 0.0001), and the second one is to dynamically adjust the learning rate by initially setting it to 0.001 and then reducing it with a factor of 0.1 when the validation loss stops improving for ten epochs. We tested three kernel sizes (3, 5, and 7), three normalization methods (batch, instance, and group), and two hidden dimensions (64 and 128). The number of residual blocks and the corresponding dilations for each block that we tested can be found in S4 Table for 10 kb and S5 Table for 5 kb. In addition, we trained another deep learning architecture named axial-attention networks [27] to compare with the ResNet that we used in DeepChIA-PET. For tuning axial-attention networks, we tested three numbers of heads (1, 2, and 4) and three numbers of blocks (2, 4, and 6). The loss function for all models is binary cross entropy. We also used different positive weights (1, 3, 6, 9, and 12) when calculating loss. The best model for blind testing is the model that achieved the lowest validation loss. All models were trained in parallel on four NVIDIA A100 GPUs; each is equipped with 40 GB of memory. The computational cost for training and blind testing can be found in the S1 Note.

### Evaluation metrics

Because the genomic distances of ground-truth ChIA-PET interactions are within a certain range, we only evaluated the pixels that are within the genomic range from 20 kb to 2 Mb. Since the ratios between the numbers of negative and positive pixels are usually larger than 500 at 10-kb resolution and 1000 at 5-kb resolution (S2 Fig), we defined five different negative pixel sets (“neg = pos”, “neg = 5pos”, “neg = 20pos”, “neg = 100pos”, and “neg = All”) for evaluation. The number of negative pixels in the first four sets for each chromosome was set to the given number (i.e., 1, 5, 20, and 100) times the number of positive pixels on this chromosome, and the negative pixels for evaluation were randomly selected from all negative pixels. Therefore, “neg = pos” means that the number of negative pixels that we used for blind testing is equal to the number of positive pixels. In the last negative set (“neg = All”), we used all negative pixels. Please note that the above-mentioned were for blind testing, for training all negative pixels were used.

For evaluating the performance of pixel-specific binary classification, we used various metrics including average precision (AP), mean area under the receiver operating characteristic (ROC) curve (AUC), and precision-recall curve [28]. The evaluation procedures were conducted at each pixel level without allowing any mismatch. In other words, the AP and AUC scores were directly calculated between the ground-truth pixels and their predicted scores. We also borrowed the metric of top accuracy from the community of protein contact map prediction [20,21]. Specifically, we selected a set of pixels with the top N predicted scores and calculated the percentage (accuracy) of ground-truth pixels that are found in the set. If N is not given a specific number in downstream analysis, top-N means that we select the top number of pixels that is equal to the ground-truth number of positive pixels for each chromosome.

## Results

### Hyperparameter selection

The testing results of hyperparameter tuning on chromosome 1 for 10-kb resolution are shown in S4 Table. The training data were generated from chromosomes 3 to X, while the validation data were extracted from chromosome 2. From the learned models with the positive weight 3, we observed that different batch sizes do affect validation loss, and batch size 16 is a better selection for either one of the two normalization methods (instance or batch). After we fixed the batch size to 16, we found that compared with 0.01 and 0.0001 using the initial learning rate 0.001 achieved a smaller validation loss. Since batch sizes matter and instance normalizations are not related to batch size, we applied batch normalization for all convolutional layers in our network. We extended the number of residual blocks to 40; each block has its dilation value for dilated convolutions (S4 Table). After increasing the hidden dimension to 128, we obtained the final optimal combination of hyperparameters that achieved the lowest validation loss: the batch size 16, the learning rate 0.001 with automatic reducing, the kernel size 3, and the positive weight 1. The training and validation loss curves that we obtained with the optimal hyperparameters are shown in S3 Fig We did not observe overfitting; instead, we obtained an almost perfect loss curve for validation, which is very similar to the loss curves from the axial attention network. The final axial-attention model that we used is the one that was trained with the number of heads equal to 4 and the number of blocks equal to 2 (S6 Table). To train the other 22 models for blind testing on chromosomes from 2 to X, we used the same hyperparameters as we obtained from testing chromosome 1. The results for hyperparameter tuning of 5-kb resolution are shown in S5 Table.

### DeepChIA-PET(Hi-C) significantly outperforms Peakachu

To make a fair comparison with Peakachu, we trained DeepChIA-PET with only Hi-C data as input. The hyperparameters for training DeepChIA-PET(Hi-C) were the same as the ones we obtained in the hyperparameter-tuning process. The main difference is that the input channel is changed from three to one. We also trained DeepChIA-PET(Hi-C) and Peakachu using the same input and ground-truth data. We used “score_chromosome” in Peakachu [17] to predict interaction probability per pixel for the testing chromosomes. The comparison between Peakachu and DeepChIA-PET(Hi-C) is to directly evaluate their abilities in identifying pixel-specific ChIA-PET interactions.

**Fig 2:**
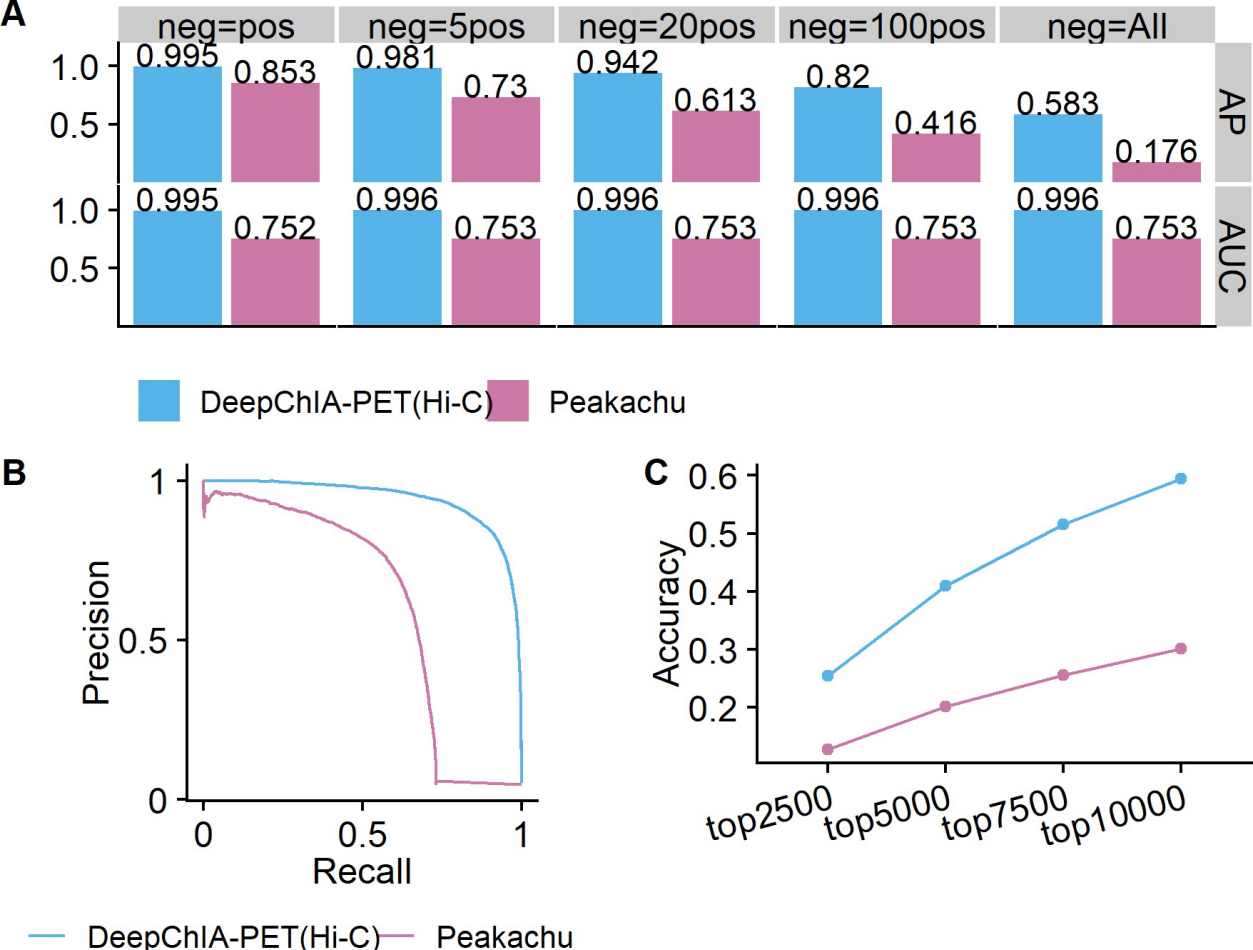
DeepChIA-PET(Hi-C) outperforms Peakachu for blindly testing CTCF ChIA-PET on chromosome 1 in GM12878 at 10-kb resolution. (A) The AP and ROC-AUC scores in terms of different numbers of negative pixels when evaluating. (B) The precision-recall curve for the number of negative pixels equaling 20 times the number of positive pixels (“neg = 20pos”). (C) The top-N accuracy where N equals four different values.

We tested two chromosomes 1 and 2, and the comparison results are shown in Figs 2, S4 and S5, respectively. With the increase of the number of negative pixels (two more negative sets tested, neg = 0.1pos and neg = 0.5pos in S4 and S5 Figs), the AP values keep reducing for both DeepChIA-PET(Hi-C) and Peakachu, whereas the AUCs have not been affected (Fig 2A). Notably, DeepChIA-PET(Hi-C) achieved higher APs than Peakachu and almost perfect AUC (0.995) compared with 0.753 for Peakachu. In addition, the PR curves shown in Fig 2B for neg = 20pos also indicate that DeepChIA-PET(Hi-C) significantly outperforms Peakachu. Moreover, we compared top accuracy scores for the two methods (Fig 2C), revealing that DeepChIA-PET(Hi-C) can successfully recover or pinpoint many more ground-truth pixels than Peakachu. We can draw the same conclusions from S5 Fig for blind testing on chromosome 2. Moreover, we tested two more window sizes (23×23 and 35×35) for Peakachu and observed that increasing window sizes does not improve its performance (S6 Fig).

When testing DeepChIA-PET on chromosome 1, the DeepChIA-PET model trained on human chromosomes 3 to X (using GM12878 data) and validated on human chromosome 2 (using GM12878 data) was used, similarly for testing on chromosome 2. The input data when making predictions is the Hi-C data of GM12878.

When testing Peakachu on chromosome 1, the Peakachu model trained (using GM12878 data) on human chromosomes 2 to X was used, similarly for testing on chromosome 2 (the model trained on chr. 1, 3, 4, 5, 6, …, and X was used). The input data when making predictions are the Hi-C data of GM12878.

### ChIP-seq data improve the performance of DeepChIA-PET

We next investigated the contribution of ChIP-seq data in DeepChIA-PET. We trained four more DeepChIA-PET models with only Hi-C as input, with only ChIP-seq as input, and with both ChIP-seq and Hi-C as input for blind testing on chromosomes 1 and 2, and the evaluation results are shown in Figs S7, S8 and 3. When testing DeepChIA-PET on chromosome 1, the DeepChIA-PET model that was trained (using GM12878 data) on human chromosomes 3 to X and validated on human chromosome 2 was used, similarly for testing on chromosome 2.

**Fig 3:**
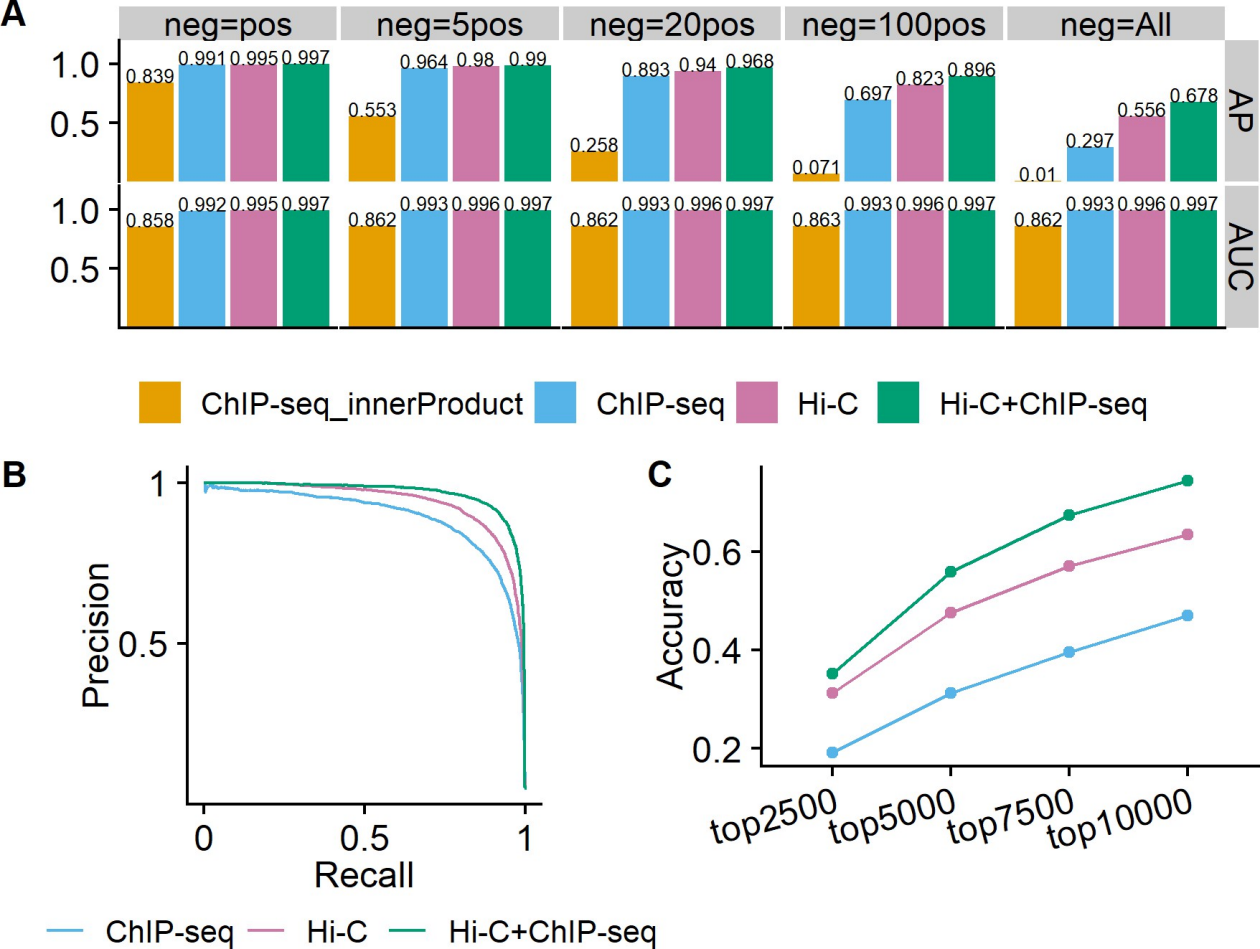
Ablation studies of the contribution of ChIP-seq. The evaluations for DeepChIA-PET with only Hi-C, only ChIP-seq, and both as input were conducted on chromosome 2 for CTCF ChIA-PET in GM12878 at 10-kb resolution. (A) The AP and ROC-AUC scores for different numbers of negative pixels. The inner product of ChIP-seq is provided as a baseline. (B) Precision-recall curve for “neg = 20pos”. (C) The top-N accuracy where N equals four different values.

As in the comparisons between DeepChIA-PET(Hi-C) and Peakachu, we calculated AP and AUC for each negative set, drew the PR curves for neg = 20pos, and obtained the top-N accuracy scores. From Fig 3, we can conclude that (1) DeepChIA-PET with both Hi-C and ChIP-seq as input consistently outperforms the other two cases in all the metrics; (2) the performance of DeepChIA-PET with Hi-C as input or DeepChIA-PET(Hi-C) is much closer to DeepChIA-PET(Hi-C and ChIA-PET) than DeepChIA-PET with only ChIP-seq as input or DeepChIA-PET(ChIP-seq); and (3) adding ChIP-seq together with Hi-C does significantly improve the performance of our classifier, and the improvements are statistically significant (S7 Fig). The same conclusions can be drawn when blind testing on chromosome 1 (S8 Fig).

In addition, we calculated the inner product of ChIP-seq as a baseline, in which the prediction of the pixel [*i*,*j*] is the inner product of two ChIP-seq vectors ([*i*-5,*i*+5] and [*j*-5,*j*+5]). The three DeepChIAPET methods perform noticeably better than the baseline (Figs 3A and S8A), especially in terms of AP. With the increase in the number of negative samples used for evaluation, the AP values of the inner product sharply reduce, indicating that the predictions from the inner product contain a lot of false positives.

### Overall performance on CTCF ChIA-PET in GM12878

**Fig 4:**
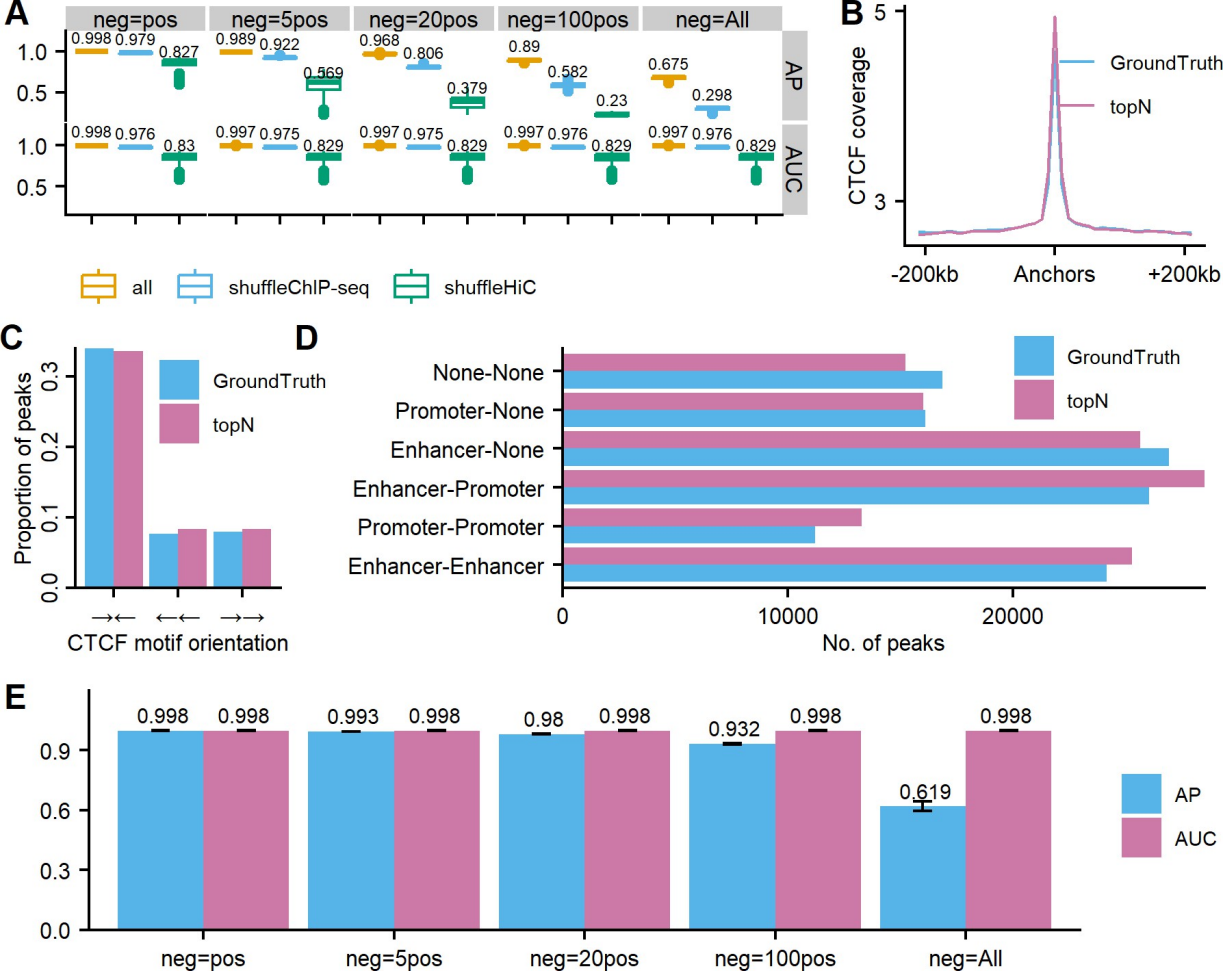
Overall performance of DeepChIA-PET for predicting CTCF ChIA-PET interactions on all testing chromosomes in GM12878 at 10-kb and 5-kb resolution. (A) The boxplots of APs and ROC-AUCs for all testing chromosomes at 10-kb resolution (mean values are added above each boxplot) with three input types: all (real input), shuffleChIP-seq (shuffling 1D ChIP-seq vector before converting to two 2D matrices), and shuffleHiC (shuffling the 2D Hi-C matrix). (B) The average CTCF coverage surrounding anchors from ground truth and our predicted top-N pixels. (C) CTCF motif orientation analysis at anchors from ground truth and our predicted top-N pixels. (D) ChIA-PET interactions between promoters and enhancers. (E) The APs and ROC-AUCs for chromosomes 1–5 at 5-kb resolution. Error bars show standard deviation (SD).

DeepChIA-PET achieved state-of-the-art performance on all testing chromosomes from chromosome 1 to the X-chromosome in GM12878 (Fig 4A) at 10-kb resolution. Specifically, we achieve almost perfect AUC (mean ≥ 0.997) for all five different negative sets, and the higher AP values also indicate that DeepChIA-PET can successfully identify positive pixels among an imbalanced pool that is heavily occupied by negatives. We separately shuffled Hi-C and ChIP-seq to investigate which feature is more important. The evaluation results are also shown in Fig 4A, suggesting that both two features play an increasingly important role in achieving high AP values with more negative pixels being considered, especially for the Hi-C feature.

In addition, we compared the performance of our residual network with the axial-attention network for predicting CTCF ChIA-PET on chromosome 1 in GM12878, and the results (S9 Fig) suggest that our residual model performs slightly better than the axial-attention model.

Next, we focused on our predicted top-N pixels to find out if these pixels have similar transcriptional properties with ground-truth pixels. We first found that the genomic regions surrounding anchors from both top-N and ground truth are enriched with CTCF (Fig 4B), which is what we expected for CTCF ChIA-PET interactions. We then found CTCF motif orientation using MotifFinder in Juicer [18] and observed that the pairs of CTCF motifs that anchor most of top-N and ground-truth pixels (> 60%) are in the convergent orientation (Fig 4C) and our top-N pixels have very similar CTCF motif orientation (convergent and tandem) with ground truth.

Moreover, we assigned regulatory elements (promoters, enhancers, or none for not finding any promoter or enhancer) to each anchor at top-N and ground-truth loci and counted the number of pixels that belong to each of the six different regulatory element combinations. The promoter and enhancer loci of GM12878 were extracted from ChromHMM segmentation in ENCODE with 10 states. We found that most of the pixels in both top-N and ground truth are related to at least one of the well-known regulatory elements (Fig 4D). Particularly, our top-N set contains more significant interactions than ground truth for the three regulatory element combinations (enhancer-enhancer, promoter-promoter, and enhancer-promoter).

The evaluation results for chromosomes 1–5 at 5-kb resolution are shown in Fig 4E. The APs and AUCs that we obtained at 5-kb resolution are as good as what we observed in Fig 4A at 10-kb resolution, indicating that we can successfully apply our method to higher resolutions.

**Fig 5:**
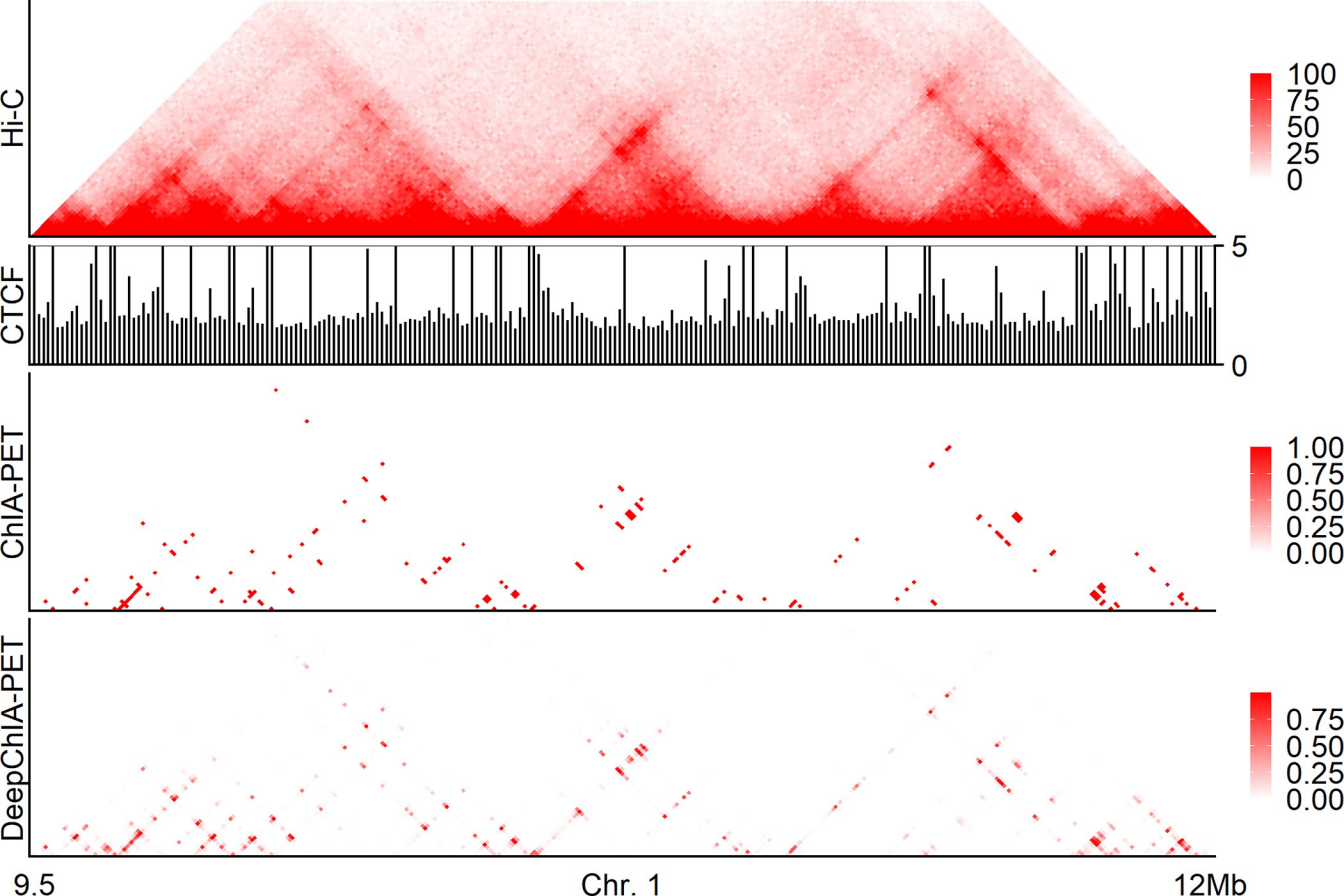
A specific example of our predictions for CTCF ChIA-PET interactions on chromosome 1 in GM12878 at 10-kb resolution. From top to bottom: the KR-normalized Hi-C, the CTCF coverage at 10-kb resolution, the heat map for ground-truth ChIA-PET interactions, and the heat map for our predicted ChIA-PET interactions.

We showed two specific examples of our predicted CTCF ChIA-PET interactions on chromosomes 1 (Fig 5) and 10 (S10 Fig) in GM12878. We observed several TADs in the heat map of KR-normalized Hi-C. We also found that the insulator-binding protein CTCF coverages are enriched not only in surrounding TAD boundary regions but also at some non-boundary loci. Moreover, we found that the anchors of ground-truth ChIA-PET interactions are usually located between the genomic regions that are enriched for the binding of CTCF, and the heat map of our predicted CTCF ChIA-PET is so accurate that it looks like a mirror image of the ground-truth heat map.

### Overall performance on CTCF ChIA-PET in HeLa

We next evaluated DeepChIA-PET on CTCF ChIA-PET in a different cell type HeLa. When testing on chromosome 1 in Hela, the model trained on human chromosomes 3 to X (using the data of GM12878) and validated on human chromosome 2 (using the data of GM12878) was used, similarly for testing on the other chromosomes. The input of the model when making predictions is the Hi-C and CTCF ChIP-seq data of Hela.

**Fig 6:**
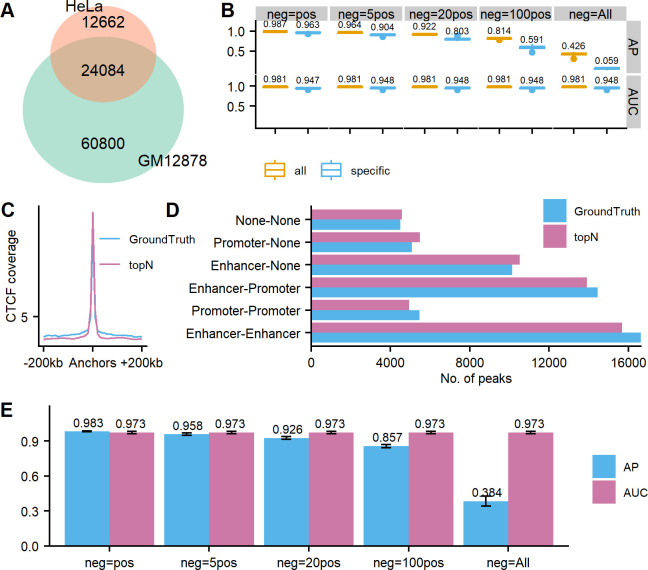
Overall performance of DeepChIA-PET for CTCF ChIA-PET predictions in a different cell type HeLa at 10-kb and 5-kb resolution. (A) Overlap in ground-truth ChIA-PET interactions between GM12878 and HeLa. (B) The boxplots of APs and ROC-AUCs for all testing chromosomes (chr.1-X) at 10-kb resolution (mean values are added above each boxplot) on all HeLa loops and HeLa-specific loops. (C) The average CTCF coverage surrounding anchors from ground truth and our predicted top-N pixels. (D) ChIA-PET interactions between promoters and enhancers. (E) The APs and ROC-AUCs for chromosomes 1–5 at 5-kb resolution. Error bars show standard deviation (SD).

We first checked the similarities of ground-truth CTCF ChIA-PET between GM12878 and HeLa. We found that the number of HeLa loops is less than half of the number of GM12878 loops and ~65.5% HeLa ChIA-PET loops were found in GM12878 (Fig 6A), indicating that HeLa ChIA-PET interactions are sparser than GM12878.

The overall performance of DeepChIA-PET for predicting CTCF ChIA-PET in HeLa at 10-kb resolution is shown in Fig 6B. Compared with the performance in GM12878, the AUC values have a slight decrease from 0.997 to 0.981, and the AP values have a noticeable drop when using all negative pixels (neg = All), which may result from the massively increasing of the number of negative pixels. We also reported the APs and AUCs for predicting HeLa-specific ChIA-PET loops, results see Fig 6B, which are a bit lower than the corresponding values for all loops. Our residual network performs better than the axial-attention network in terms of AP (S11 Fig). The anchors from ground truth and the top-N set in HeLa are enriched for CTCF (Fig 6C), and the two sets (top-N and ground truth) have very similar loop distributions for the six different regulatory element combinations (Fig 6D). As in GM12878, the promoter and enhancer loci of HeLa were extracted from ChromHMM segmentation in ENCODE. The performance of DeepChIA-PET at 5-kb resolution (Fig 6E) is comparable to the ones at lower resolution (10 kb).

**Fig 7:**
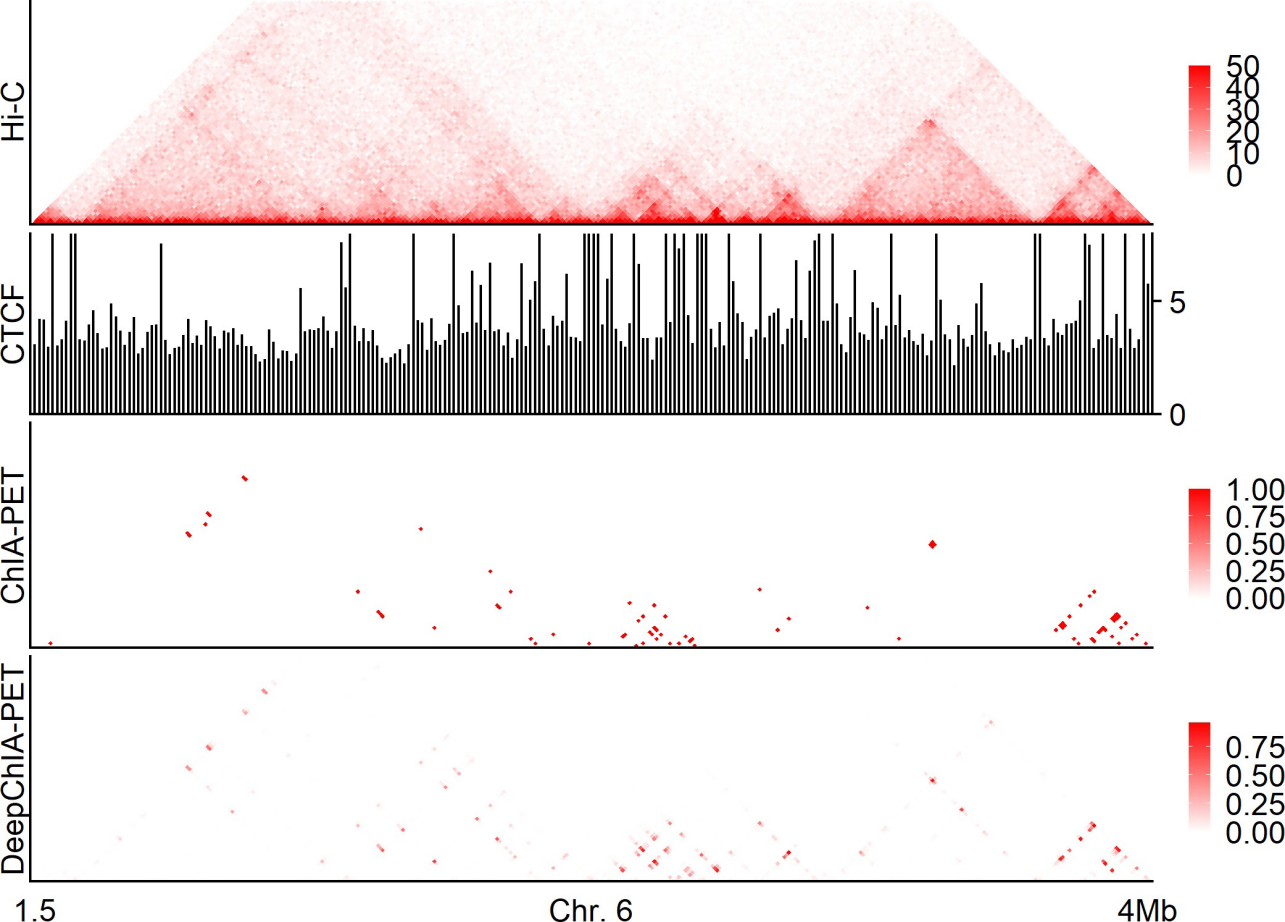
A specific example of our predictions for CTCF ChIA-PET on chromosome 6 in HeLa at 10-kb resolution. From top to bottom: KR-normalized Hi-C, CTCF coverage at 10-kb resolution, ground-truth ChIA-PET, and our predicted ChIA-PET.

A specific example of our predictions on chromosome 6 in HeLa is shown in Fig 7, we can make the same conclusions as we made from Figs 5 and S10. In summary, DeepChIA-PET that was trained with CTCF ChIA-PET in GM12878 can accurately predict CTCF ChIA-PET in HeLa.

### Overall performance on RNAPII ChIA-PET in GM12878 and HeLa

Since ChIA-PET experiments can be conducted on different DNA-binding proteins, we used DeepChIA-PET to predict RNAPII ChIA-PET in GM12878 and HeLa. When testing on chromosome 1 in GM12878 and Hela, the model trained (using GM12878 data) on human chromosomes 3 to X and validated (using GM12878 data) on human chromosome 2 (the largest chromosome after excluding chr.1) was used, similarly for testing on the other chromosomes. The input of the model when making predictions are the Hi-C and RNAPII ChIP-seq data of GM12878 and Hela, respectively. The execution time for predicting RNAPII ChIA-PET of GM12878 at 10-kb resolution is shown in S12 Fig.

**Fig 8:**
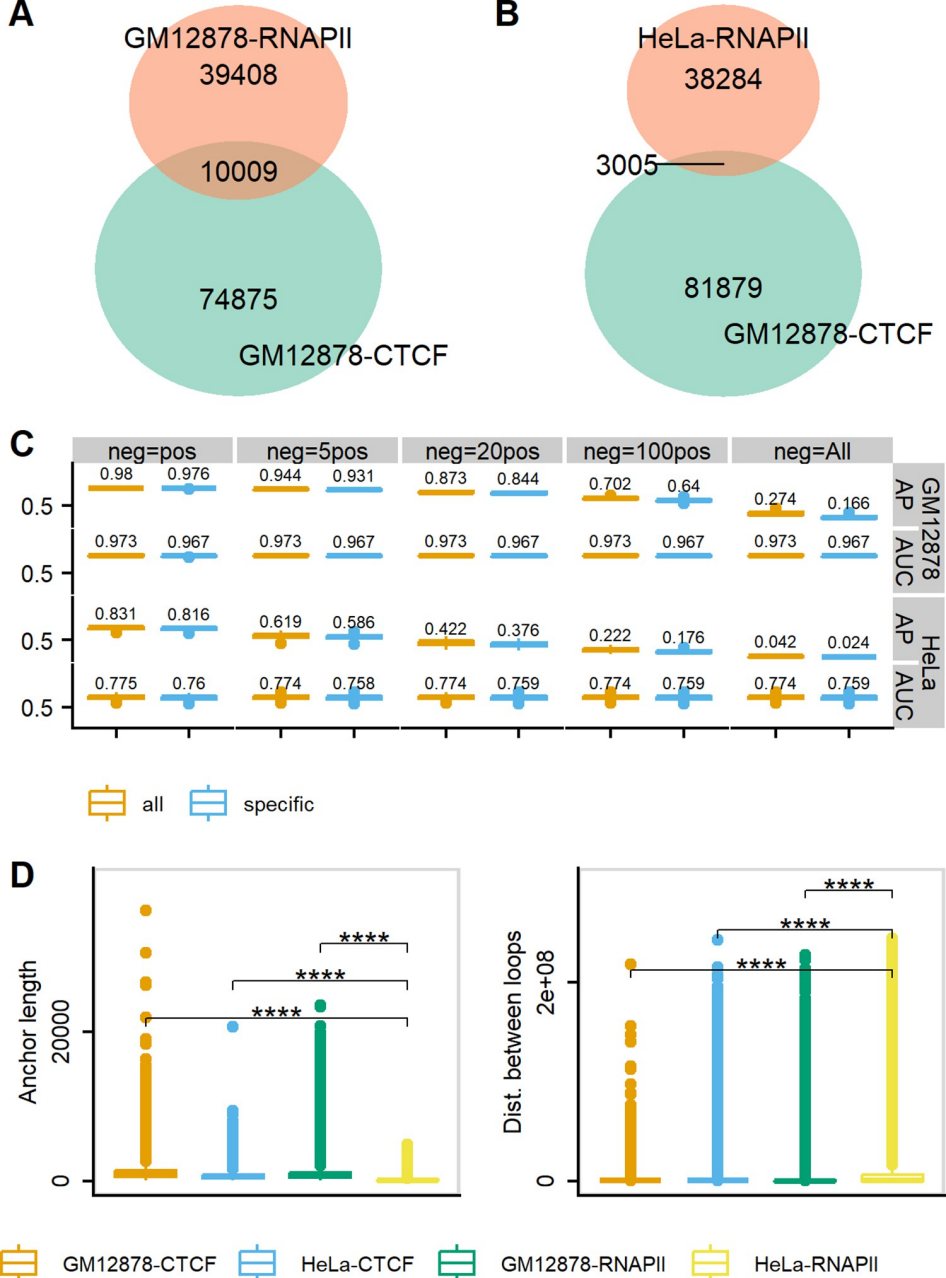
Overall performance of DeepChIA-PET on predicting RNAPII ChIA-PET in GM12878 and HeLa. The overlaps in ground-truth pixel-wise ChIA-PET interactions between CTCF GM12878 and RNAPII GM12878 are shown in (A), and between CTCF GM12878 and RNAPII HeLa are shown in (B). (C) The boxplots of APs and ROC-AUCs for all testing chromosomes in both GM1278 and HeLa on all and specific loops. (D) RNAPII ChIA-PET interactions for HeLa are significantly different from either the RNAPII ChIA-PET in GM12878 or the CTCF ChIA-PET in both GM12878 and HeLa by having shorter anchor lengths and longer genomic distances between interactions. Mean comparisons were conducted with the student’s t-Test (****: p < = 0.0001).

We first compared the similarities between CTCF ChIA-PET of GM12878 and RNAPII ChIA-PET in GM12878 and HeLa (Fig 8A and 8B) and found that RNAPII ChIA-PET does not share most of its loops with CTCF ChIA-PET even though the cell types are the same.

The genome-wide AP and AUC values of DeepChIA-PET at 10-kb resolution are shown in Fig 8C. For predicting RNAPII ChIA-PET of GM12878, our tool achieved a higher AUC of 0.973, and the AP performs well except when we used all negative pixels (neg = All) for evaluation. However, for predicting RNAPII ChIA-PET of HeLa, we achieved an AUC of 0.771, and the AP values keep reducing with the increase of negative pixels used for evaluation. The reason that DeepChIA-PET did not perform well in predicting RNAPII ChIA-PET of HeLa may be that RNAPII ChIA-PET loops of HeLa have shorter anchor lengths and longer genomic distances (Fig 8D), which is significantly different from what we found from CTCF ChIA-PET of GM12878. The accuracy of DeepChIA-PET when predicting all HeLa loops is higher than when predicting HeLa-specific loops (Fig 8C). The overall performance of DeepChIA-PET at 5-kb resolution is comparable to that at 10-kb resolution (S13 Fig). Together, DeepChIA-PET can accurately predict RNAPII ChIA-PET of GM12878, but not perform well in RNAPII ChIA-PET of HeLa.

### Overall performance on RAD21 ChIA-PET in GM12878 and K562 from different experiments

The CTCF and RNAPII ChIA-PET data that we used for evaluation are from the same study [3]. In this section, we evaluated DeepChIA-PET in predicting RAD21 ChIA-PET interactions, which were obtained from different experiments [4]. When testing on chromosome 1 in GM12878 and K562, the model trained (using GM12878 data) on human chromosomes 3 to X and validated (using GM12878 data) on human chromosome 2 was used, similarly for testing on the other chromosomes. The input of the model when making predictions is the Hi-C and RAD21 ChIP-seq data of GM12878 and K562, respectively.

**Fig 9:**
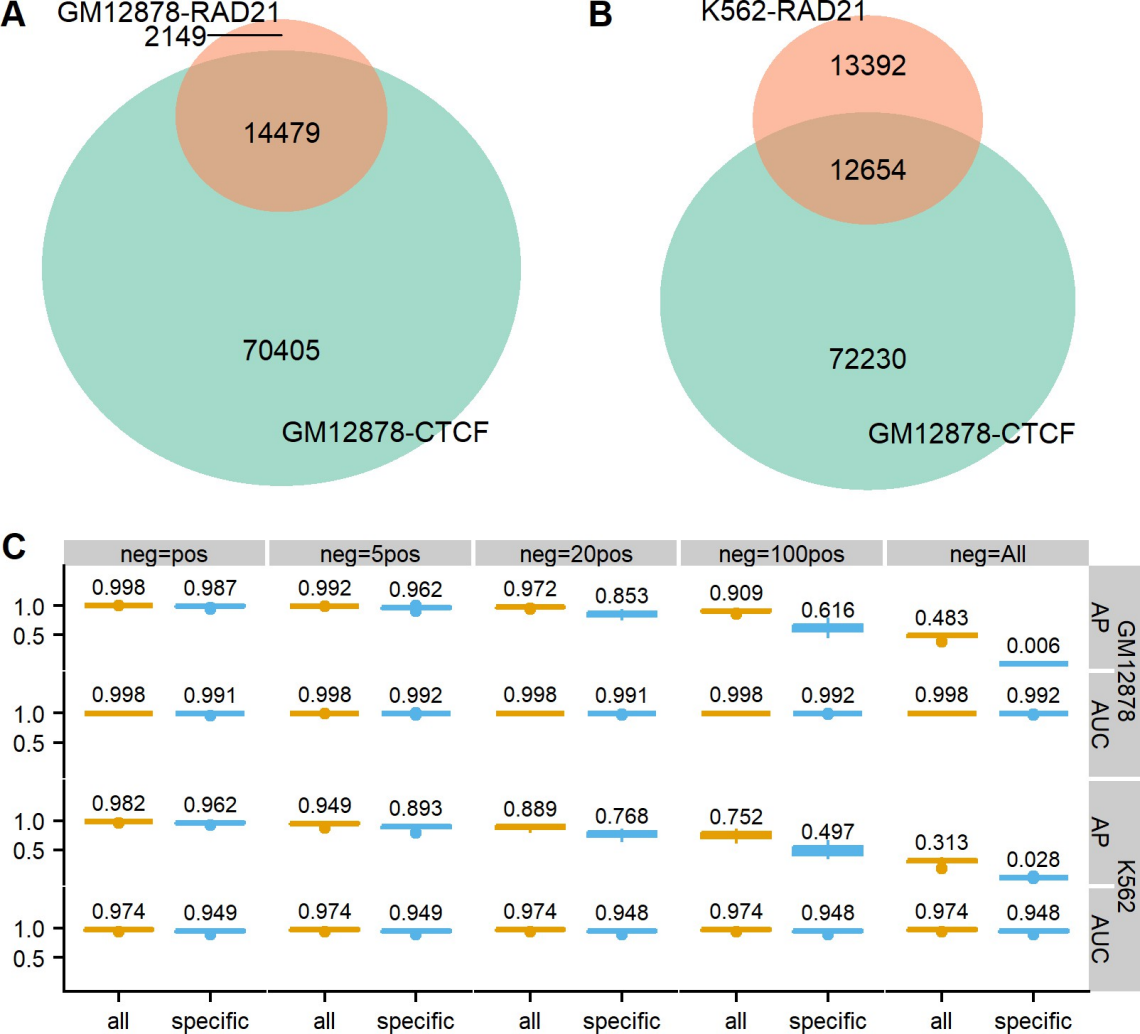
Overall performance on predicting RAD21 ChIA-PET in both GM12878 and K562 at 10-kb resolution. The overlaps in ground-truth pixel-wise ChIA-PET interactions between CTCF GM12878 and RAD21 GM12878 are shown in (A), and between CTCF GM12878 and RAD21 K562 are shown in (B). (C) The boxplots of APs and ROC-AUCs for all testing chromosomes in both GM1278 and K562 on all and specific loops. Note: since the KR-normalized Hi-C matrix for chromosome 9 in K562 is empty, the evaluation results shown in (C) for K562 do not include the testing scores for chromosome 9.

As before, we first compared the similarities of ChIA-PET loops between CTCF of GM12878 and RAD21 of GM12878, and between CTCF of GM12878 and RAD21 of K562. We found ~87.1% of RAD21 loops in GM12878 are shared with CTCF loops of GM12878 (Fig 9A) and ~48.6% of RAD21 loops in K562 are also detected in CTCF loops of GM12878 (Fig 9B). Therefore, the two RAD21 ChIA-PET datasets are more similar to CTCF ChIA-PET of GM12878 than between the two RANPII ChIA-PET loop sets.

We reported AP and ROC-AUC scores of DeepChIA-PET for predicting all and specific RAD21 ChIA-PET of GM12878 and RAD21 ChIA-PET of K562 (Fig 9C) at 10-kb resolution. DeepChIA-PET achieved almost perfect ROC-AUCs (0.998 for GM12878 and 0.975 for K562) and also obtained high AP values (0.972 for GM12878 and 0.891 for K562) when neg = 20pos. We also reported different top-N accuracy scores obtained on all testing chromosomes from chromosome 1 to the X-chromosome (S14 Fig) and found that the more top predicted pixels that we considered for evaluations, the higher top-N accuracy we achieved, which can be used as a guide when deciding on the number of top predicted interactions to be used in applications. Based on these evaluations, we would recommend starting with the top 15,000 predictions as the value of the parameter N since most of the best performances were achieved with this value. However, other values of N also resulted in good performances based on our evaluation, so the value of the parameter N may be data-dependent. We reported AP and AUC scores for predicting RAD21 ChIA-PET at 5-kb resolution in S15 Fig, and the similarly successful scores as in 10-kb resolution indicate that increasing resolution does not drop the accuracy of our method.

### Comparison between CTCF ChIA-PET and Hi-C peaks

**Fig 10:**
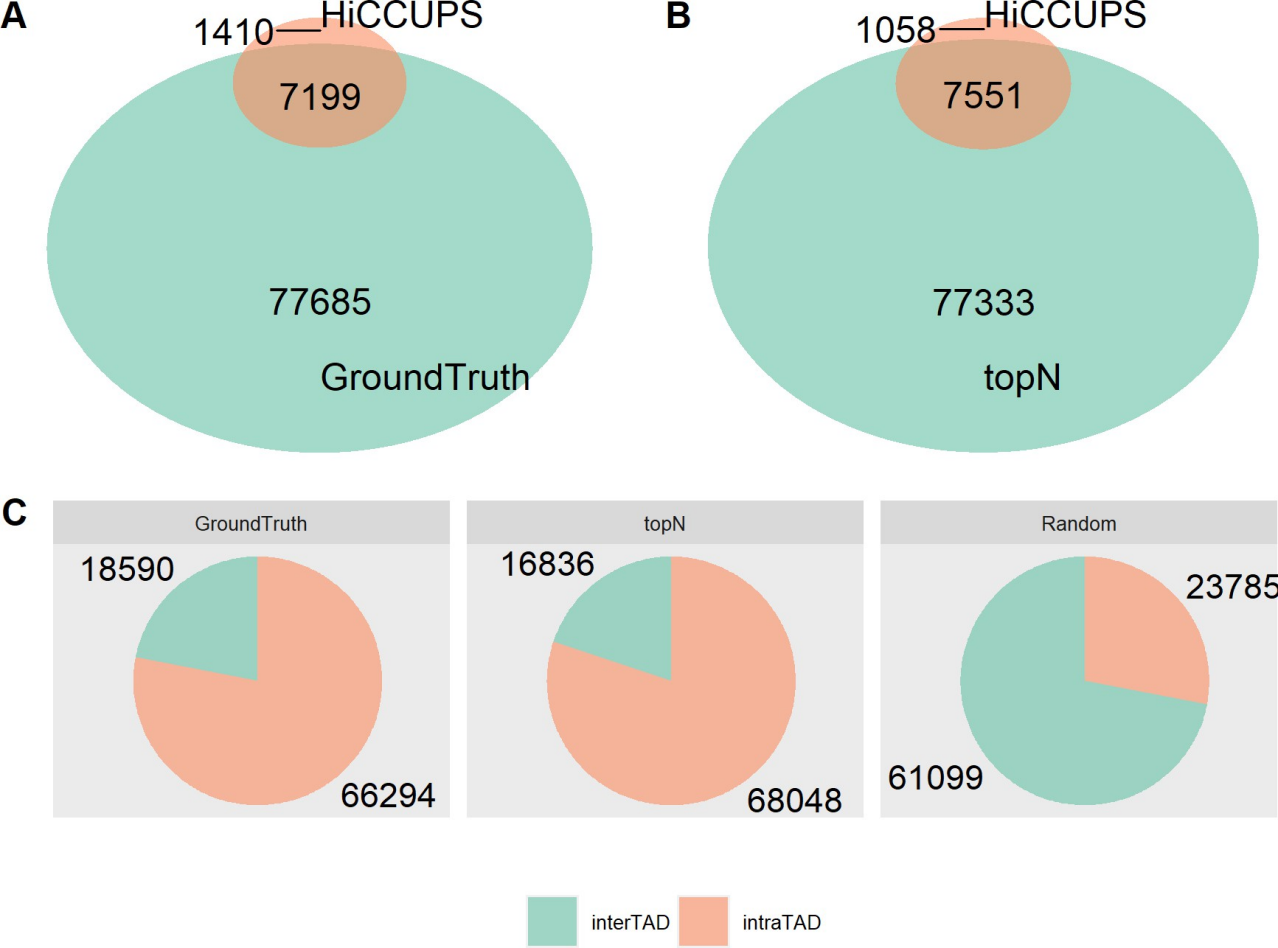
Genome-wide overlaps between Hi-C peaks called by HiCCUPS and ground-truth CTCF ChIA-PET interactions (A), and between Hi-C peaks called by HiCCUPS and our predicted top-N CTCF ChIA-PET interactions (B) in GM12878. When counting the accordant pixels between ChIA-PET and Hi-C peaks, we allow ± 1 bin mismatch. (C) Compared with randomly selected pixels, most of the ground truth and our predicted top-N CTCF ChIA-PET interactions are found within TADs.

The anchors of Hi-C peaks/loops called on Hi-C by HiCCUPS have been typically found at TAD boundaries and CTCF binding sites [8]. We detected 8609 Hi-C peaks on Hi-C contact matrices of GM12878 at 10-kb resolution. We reported the overlaps between Hi-C peaks and ground-truth CTCF ChIA-PET interactions of GM12878 (Fig 10A), and between Hi-C peaks and our top-N predicted set (Fig 10B). It is observed that most of the Hi-C peaks can also be found in the two CTCF ChIA-PET loop sets: 83.6% Hi-C peaks are shared with ground-truth ChIA-PET, whereas 87.7% Hi-C peaks are also found in top-N, indicating that DeepChIA-PET can select Hi-C peaks as potential ChIA-PET loops.

In addition, we explored the relationships between CTCF ChIA-PET interactions and TADs. We found that compared with 28% of randomly selected pixels, more than 78% of ground-truth and 80% of predicted top-N CTCF ChIA-PET interactions are found within TADs (Fig 10C).

### Predicting CTCF ChIA-PET in HMEC and NHEK

In the last, we tried to predict CTCF ChIA-PET of the cell types that have Hi-C and ChIP-seq data available but not the ChIA-PET data. We used two cell types (HMEC and NHEK) and predicted their CTCF ChIA-PET loops at 10-kb resolution.

When testing on chromosome 1 in HMEC and NHEK, the model trained (using GM12878 data) on human chromosomes 3 to X and validated on human chromosome 2 (using GM12878 data) was used, similarly for testing on the other chromosomes. The input of the model when making predictions is the Hi-C and CTCF ChIP-seq data of HMEC and NHEK, respectively.

We first found that for both cell types more than half of the top-N loops are specific (S16A Fig), where N is the number of CTCF ChIA-PET in GM12878. We then compared these specific loops with their corresponding Hi-C peaks called by HiCCUPS and observed that 42.26% of HMEC (S16B Fig) and 38.7% of NHEK (S16C Fig) Hi-C peaks are found in their specific loops. Finally, we demonstrated that compared with GM12878, the HMEC-specific (S16D Fig) and NHEK-specific (S16E Fig) anchors are more enriched for their own CTCF peaks. These can indicate that the ChIA-PET interactions that DeepChIA-PET predicts fit the other biological findings although stringent benchmarking is not performed as we do not have the experimental ChIA-PET data for these two cell types.

## Discussion

In this study, we present DeepChIA-PET, a supervised deep-learning method for predicting ChIA-PET from Hi-C and ChIP-seq data at 10-kb and 5-kb resolution. Our evaluation results indicate that DeepChIA-PET with only Hi-C as input significantly outperforms Peakachu. The ablation studies prove that ChIP-seq data as input contributes to the classification task of DeepChIA-PET. For predicting CTCF ChIA-PET in GM12878 and HeLa, DeepChIA-PET can achieve ROC-AUCs of 0.997 and 0.973, and our predicted top-N loops have very similar patterns of CTCF motif orientation with ground-truth ChIA-PET interactions in GM12878. The regulatory elements are widely found at most of the anchors from our top-N loops, and the distributions of different regulatory element interactions are very similar to those from the ground truth. We also reported that DeepChIA-PET can be used to accurately predict non-CTCF ChIA-PET interactions, including RNAPII ChIA-PET of GM12878, RAD21 ChIA-PET of GM12878, and RAD21 ChIA-PET of K562, even though these ChIA-PET interactions are usually different from CTCF ChIA-PET of GM12878 in terms of the number of total and overlapping interactions. We compared CTCF ChIA-PET interactions with Hi-C peaks and TADs and found that our top-N loops have more common pixels with Hi-C peaks than ground truth and the number of our top-N loops that are located within TADs is more than ground truth.

## Supporting information

S1 Note The computational cost. (DOCX)

S1 Table The source of Hi-C datasets. (DOCX)

S2 Table The source of ChIP-seq datasets. (DOCX)

S3 Table The source of ChIA-PET datasets. (DOCX)

S4 Table Results for hyperparameter tuning of residual networks at 10-kb resolution. The batch size for validation data was set to one for all models. Learning rates followed by “(↓)” indicate that we reduced the learning rate by a factor of 0.1 when validation loss stops improving. Positive weights followed by a * mean that we only used this positive weight when calculating training loss; if there is no * we used the positive weight for calculating both training and validation losses. Therefore, we can compare validation loss between models with the same positive weight without having * or models with positive weights followed by a *. All models are trained for blind testing on chromosome 1, which means we extract training data from chromosome 3 up to X and validation data from chromosome 2.
(DOCX)

S5 Table Results for hyperparameter tuning of residual networks at 5-kb resolution. The number 2 in Group Normalization is the number of groups we used.
(DOCX)

S6 Table Results for hyperparameter tuning of axial attention networks at 10-kb resolution. The training data are extracted from chromosome 3 up to X and validation data from chromosome 2 for predicting CTCF ChIA-PET in GM12878.
(DOCX)

S1 Fig For CTCF ChIA-PET interactions in GM12878, we find (A) almost all anchors’ genomic lengths are less than 10 kb, and (B) almost all ChIA-PET interactions are within 2 Mb.
(TIFF)

S2 Fig The number of positive and negative pixels for each chromosome and the ratios between negative and positive at resolutions of 10 kb and 5 kb. The range of genomic distances we cared about is from 2 to 200 bins for 10 kb and from 2 to 400 for 5 kb, that is, 20 kb to 2 Mb. The ChIA-PET data are from CTCF of GM12878.
(TIFF)

S3 Fig Binary cross entropy loss curves for training and validation of two deep networks (ConvNet and axial attention) at 10-kb resolution. The learned model was trained for blindly testing on chromosome 1. The validation data were extracted from chromosome 2. The training data were generated from the rest of the chromosome from 3 to X.
(TIFF)

S4 Fig The proportion of negative pixels affects final evaluation performance. DeepChIA-PET(Hi-C) outperforms Peakachu for blind testing CTCF ChIA-PET on chromosome 1 in GM12878 in terms of AP and AUC at 10-kb resolution.
(TIFF)

S5 Fig DeepChIA-PET(Hi-C) outperforms Peakachu for blindly testing CTCF ChIA-PET on chromosome 2 in GM12878 at 10-kb resolution. (A) The AP and AUC results for different number of negative pixels used for evaluating. (B) Precision-recall curve for the number of negative pixels equal to 20 times the number of positive pixels. (C) The top-N accuracy where N equals four different values.
(TIFF)

S6 Fig Peakachu performs best when using a window size of 11×11. The AP and AUC results for the three window sizes are from blindly testing CTCF ChIA-PET on chromosomes 1 and 2 in GM12878 at 10-kb resolution.
(TIFF)

S7 Fig The improvements (ChIP-seq vs Hi-C, ChIP-seq vs Hi-C+ChIP-seq, and Hi-C vs Hi-C+ChIP-seq) are statistically significant with student’s t-test. Each negative set is randomly generated 10 times. ****: p-value < = 0.0001. The evaluation for DeepChIA-PET with only Hi-C, only ChIP-seq, and both as input is conducted on chromosome 2 for CTCF ChIA-PET in GM12878 at 10-kb resolution.
(TIFF)

S8 Fig Adding ChIP-seq data as input improves the classification performance. The evaluation for DeepChIA-PET with only Hi-C, only ChIP-seq, and both as input is conducted on chromosome 1 for CTCF ChIA-PET in GM12878 at 10-kb resolution. (A) The AP and AUC results for different number of negative pixels used for evaluating. The inner product of ChIP-seq is provided as a baseline. (B) Precision-recall curve for the number of negative pixels equal to 20 times number of positive pixels. (C) The top-N accuracy where N equals four different values.
(TIFF)

S9 Fig The convolutional network performs slightly better than the axial-attention network. The AP and AUC values are calculated for testing CTCF ChIA-PET on chromosome 1 in GM12878 at 10-kb resolution.
(TIFF)

S10 Fig A specific example of our predictions for CTCF ChIA-PET interactions on chromosome 10 in GM12878 at 10-kb resolution. From top to bottom: KR-normalized Hi-C, CTCF, ground-truth ChIA-PET, and our predicted ChIA-PET.
(TIFF)

S11 Fig The convolutional network performs markedly better than the axial-attention network. The AP and AUC values are calculated for testing CTCF ChIA-PET on chromosome 1 in HeLa at 10-kb resolution.
(TIFF)

S12 Fig Execution time for predicting RNAPII ChIA-PET of GM12878 at 10-kb resolution. For predicting each chromosome, we set the batch size to 2 and used four NVIDIA A100 GPUs (each equipped with 40GB memory) in parallel.
(TIFF)

S13 Fig DeepChIA-PET performances for predicting RNAPII ChIA-PET of GM12878 (A) and HeLa (B) on chromosomes 1–5 at 5-kb resolution. Error bars show standard deviation (SD) with n = 5.
(TIFF)

S14 Fig The top-N accuracy scores of DeepChIA-PET for predicting RAD21 ChIA-PET of GM12878 (A) and K562 (B) at 10-kb resolution. Both (A) and (B) were generated on all testing chromosomes (mean values were added above each boxplot). Note: since the Hi-C matrix for chromosome 9 in K562 is empty, the evaluation results shown in (B) do not include the accuracy score for chromosome 9.
(TIFF)

S15 Fig DeepChIA-PET performances for predicting RAD21 ChIA-PET of GM12878 (A) and K562 (B) on chromosomes 1–5 at 5-kb resolution. Error bars show standard deviation (SD) with n = 5.
(TIFF)

S16 Fig Results for predicting CTCF ChIA-PET of HMEC and NHEK at 10-kb resolution. (A) compared with CTCF ChIA-PET of GM12878, the number of common and specific loops for HMEC and NHEK. For each chromosome, the top-N loops are considered. Here N indicates the number of loops in GM12878. Genome-wide overlaps between Hi-C peaks called by HiCCUPS and HMEC-specific CTCF ChIA-PET interactions (B), and between Hi-C peaks called by HiCCUPS and NHEK-specific CTCF ChIA-PET interactions (C). When counting the accordant pixels between ChIA-PET and Hi-C peaks, we allow ± 1 bin mismatch. The specific anchors from HMEC (D) and NHEK (E) are enriched for CTCF peaks compared with GM12878.
(TIFF)
