# Supplementary material for: DeepChIA-PET: Accurately predicting ChIA-PET from Hi-C and ChIP-seq with deep dilated networks
Source: PLoS Comput Biol. 2023 Jul 13;19(7):e1011307. doi: 10.1371/journal.pcbi.1011307 (PMC10368233; doi:10.1371/journal.pcbi.1011307)
Supplement: S1 Note — (DOCX) [file pcbi.1011307.s001.docx]

**Computational cost**

For training our residual networks at 10-kb resolution with the batch size of 16, it would take about 24h when using four NVIDIA A100 GPUs (each equipped with 40GB memory) in parallel. We used about 24GB of memory for each GPU during training.

For prediction, we set the batch size to two and used four NVIDIA A100 GPUs (each equipped with 40GB memory) in parallel. The memory requirement for a GPU is about 12.5 GB, when the batch size is set to two. The detailed execution time for predicting RNAPII ChIA-PET of each chromosome in GM12878 is shown in Fig. S12.
