# Supplementary material for: DeepChIA-PET: Accurately predicting ChIA-PET from Hi-C and ChIP-seq with deep dilated networks
Source: PLoS Comput Biol. 2023 Jul 13;19(7):e1011307. doi: 10.1371/journal.pcbi.1011307 (PMC10368233; doi:10.1371/journal.pcbi.1011307)
Supplement: S1 Table — (DOCX) [file pcbi.1011307.s002.docx]

**S1 Table.** The source of Hi-C datasets.

| Cell type | Source |
| --- | --- |
| GM12878 | https://hicfiles.s3.amazonaws.com/hiseq/gm12878/in-situ/primary.hic |
| HeLa | https://hicfiles.s3.amazonaws.com/hiseq/hela/in-situ/combined.hic |
| K562 | https://hicfiles.s3.amazonaws.com/hiseq/k562/in-situ/combined.hic |
| HMEC | https://hicfiles.s3.amazonaws.com/hiseq/hmec/in-situ/combined.hic |
| NHEK | https://hicfiles.s3.amazonaws.com/hiseq/nhek/in-situ/combined.hic |
