# Supplementary material for: DeepChIA-PET: Accurately predicting ChIA-PET from Hi-C and ChIP-seq with deep dilated networks
Source: PLoS Comput Biol. 2023 Jul 13;19(7):e1011307. doi: 10.1371/journal.pcbi.1011307 (PMC10368233; doi:10.1371/journal.pcbi.1011307)
Supplement: S2 Table — (DOCX) [file pcbi.1011307.s003.docx]

**Table S2.** The source of ChIP-seq datasets.

| ChIP-seq | Cell type | Source |
| --- | --- | --- |
| CTCF | GM12878 | http://hgdownload.cse.ucsc.edu/goldenPath/hg19/encodeDCC/wgEncodeBroadHistone/wgEncodeBroadHistoneGm12878CtcfStdSig.bigWig  https://hgdownload.cse.ucsc.edu/goldenPath/hg19/encodeDCC/wgEncodeBroadHistone/wgEncodeBroadHistoneGm12878CtcfStdPk.broadPeak.gz |
|  | HeLa | http://hgdownload.cse.ucsc.edu/goldenPath/hg19/encodeDCC/wgEncodeBroadHistone/wgEncodeBroadHistoneHelas3CtcfStdSig.bigWig |
|  | HMEC | https://hgdownload.cse.ucsc.edu/goldenPath/hg19/encodeDCC/wgEncodeBroadHistone/wgEncodeBroadHistoneHmecCtcfStdSig.bigWig  https://hgdownload.cse.ucsc.edu/goldenPath/hg19/encodeDCC/wgEncodeBroadHistone/wgEncodeBroadHistoneHmecCtcfStdPk.broadPeak.gz |
|  | NHEK | https://hgdownload.cse.ucsc.edu/goldenPath/hg19/encodeDCC/wgEncodeBroadHistone/wgEncodeBroadHistoneNhekCtcfStdSig.bigWig  https://hgdownload.cse.ucsc.edu/goldenPath/hg19/encodeDCC/wgEncodeBroadHistone/wgEncodeBroadHistoneNhekCtcfStdPk.broadPeak.gz |
| RNAPII | GM12878 | http://hgdownload.cse.ucsc.edu/goldenPath/hg19/encodeDCC/wgEncodeSydhTfbs/wgEncodeSydhTfbsGm12878Pol2IggmusSig.bigWig |
|  | HeLa | http://hgdownload.cse.ucsc.edu/goldenPath/hg19/encodeDCC/wgEncodeSydhTfbs/wgEncodeSydhTfbsHelas3Pol2StdSig.bigWig |
| RAD21 | GM12878 | http://hgdownload.cse.ucsc.edu/goldenPath/hg19/encodeDCC/wgEncodeSydhTfbs/wgEncodeSydhTfbsGm12878Rad21IggrabSig.bigWig |
|  | K562 | http://hgdownload.cse.ucsc.edu/goldenPath/hg19/encodeDCC/wgEncodeSydhTfbs/wgEncodeSydhTfbsK562Rad21StdSig.bigWig |
