# Supplementary material for: DeepChIA-PET: Accurately predicting ChIA-PET from Hi-C and ChIP-seq with deep dilated networks
Source: PLoS Comput Biol. 2023 Jul 13;19(7):e1011307. doi: 10.1371/journal.pcbi.1011307 (PMC10368233; doi:10.1371/journal.pcbi.1011307)
Supplement: S3 Table — (DOCX) [file pcbi.1011307.s004.docx]

**S3 Table.** The source of ChIA-PET datasets.

| ChIA-PET | Source |
| --- | --- |
| GM12878-CTCF | GEO, GSM1872886 |
| GM12878-RNAPII | GEO, GSM1872887 |
| HeLa-CTCF | GEO, GSM1872888 |
| HeLa-RANPII | GEO, GSM1872889 |
| GM12878-RAD21 | Table S1 from [1]. |
| K562-RAD21 |  |

**Reference**

1. Heidari N, Phanstiel DH, He C, Grubert F, Jahanbani F, Kasowski M, et al. Genome-wide map of regulatory interactions in the human genome. Genome Res. 2014;24(12):1905-17. <https://doi.org/10.1101/gr.176586.114> PMID: 25228660
