# Supplementary material for: DeepChIA-PET: Accurately predicting ChIA-PET from Hi-C and ChIP-seq with deep dilated networks
Source: PLoS Comput Biol. 2023 Jul 13;19(7):e1011307. doi: 10.1371/journal.pcbi.1011307 (PMC10368233; doi:10.1371/journal.pcbi.1011307)
Supplement: S4 Table — The batch size for validation data was set to one for all models. Learning rates followed by “(↓)” indicate that we reduced the learning rate by a factor of 0.1 when validation loss stops improving. Positive weights followed by a * mean that we only used this positive weight when calculating training loss; if there is no * we used the positive weight for calculating both training and validation losses. Therefore, we can compare validation loss between models with the same positive weight without having * or models with positive weights followed by a *. All models are trained for blind testing on chromosome 1, which means we extract training data from chromosome 3 up to X and validation data from chromosome 2. (DOCX) [file pcbi.1011307.s005.docx]

**S4 Table.** Results for hyperparameter tuning of residual networks at 10-kb resolution. The batch size for validation data was set to one for all models. Learning rates followed by “($\downarrow$)” indicate that we reduced the learning rate by a factor of 0.1 when validation loss stops improving. Positive weights followed by a * mean that we only used this positive weight when calculating training loss; if there is no * we used the positive weight for calculating both training and validation losses. Therefore, we can compare validation loss between models with the same positive weight without having * or models with positive weights followed by a *. All models are trained for blind testing on chromosome 1, which means we extract training data from chromosome 3 up to X and validation data from chromosome 2.

| Mid | Batch size | Learning rate | Kernel size | Norm | No. of residual blocks | Dilation for residual blocks | Hidden dimension | Positive weight | Validation loss |
| --- | --- | --- | --- | --- | --- | --- | --- | --- | --- |
| 1 | 4 | 0.001 | 5 | Instance | 6 | [1,2,1,4,1,1] | 64 | 3 | 0.00932 |
|  | 8 | 0.001 | 5 | Instance | 6 |  | 64 | 3 | 0.00913 |
|  | 16 | 0.001 | 5 | Instance | 6 |  | 64 | 3 | 0.0091 |
|  | 32 | 0.001 | 5 | Instance | 6 |  | 64 | 3 | 0.01077 |
|  | 16 | 0.01 | 5 | Instance | 6 |  | 64 | 3 | 0.01005 |
|  | 16 | 0.0001 | 5 | Instance | 6 |  | 64 | 3 | 0.09291 |
| 2 | 8 | 0.001 | 5 | Batch | 6 |  | 64 | 3 | 0.00939 |
|  | 16 | 0.001 | 5 | Batch | 6 |  | 64 | 3 | 0.00932 |
| 3 | 16 | 0.001 | 5 | Instance | 10 | [1,2,1,4,1,8,1,16,1,1] | 64 | 3 | 0.00904 |
| 4 | 16 | 0.001 | 5 | Instance | 14 | [1,2,1,4,1,8,  1,16,1,32,1,64,1,1] | 64 | 3 | 0.00901 |
| 5 | 16 | 0.001 ($\downarrow$) | 5 | Batch | 14 |  | 64 | 3 | 0.0088 |
|  | 32 | 0.001 ($\downarrow$) | 5 | Batch | 14 |  | 64 | 3 | 0.00909 |
|  | 64 | 0.001 ($\downarrow$) | 5 | Batch | 14 |  | 64 | 3 | 0.0099 |
| 6 | 16 | 0.001 ($\downarrow$) | 5 | Batch | 20 | [1,1,2,1,1,4,1,1,8,1,  1,16,1,1,32,1,1,64,1,1] | 64 | 3 | 0.00897 |
| 7 | 16 | 0.001 ($\downarrow$) | 3 | Batch | 14 | [1,2,1,4,1,8,  1,16,1,32,1,64,1,1] | 64 | 3 | 0.00875 |
|  | 16 | 0.001 ($\downarrow$) | 3 | Batch | 14 |  | 64 | 1 | 0.0046 |
| 8 | 16 | 0.001 ($\downarrow$) | 3 | Batch | 20 | [1,1,2,1,1,4,1,1,8,1,  1,16,1,1,32,1,1,64,1,1] | 64 | 3 | 0.00883 |
|  | 16 | 0.001 ($\downarrow$) | 3 | Batch | 20 |  | 64 | 6 | 0.01275 |
|  | 16 | 0.001 ($\downarrow$) | 3 | Batch | 20 |  | 64 | 12 | 0.01891 |
| 9 | 16 | 0.001 ($\downarrow$) | 3 | Batch | 20 |  | 128 | 6 | 0.01324 |
| 10 | 16 | 0.001 ($\downarrow$) | 3 | Batch | 40 | [1,1,2,1,1,4,1,1,8,  1,1,16,1,1,32,1,1,64,  1,1,1,1,1,1,1,1,1,1,1,1,  1,1,1,1,1,1,1,1,1,1] | 64 | 6 | 0.01317 |
| 11 | 16 | 0.001 ($\downarrow$) | 3 | Batch | 40 |  | 128 | 6 | 0.01319 |
| 12 | **16** | **0.001 (**$\boldsymbol{\downarrow}$**)** | **3** | **Batch** | **40** |  | **128** | **1*** | **0.00458** |
|  | 16 | 0.001 ($\downarrow$) | 3 | Batch | 40 |  | 128 | 3* | 0.00479 |
|  | 16 | 0.001 ($\downarrow$) | 3 | Batch | 40 |  | 128 | 6* | 0.00625 |
|  | 16 | 0.001 ($\downarrow$) | 3 | Batch | 40 |  | 128 | 9* | 0.00552 |
| 13 | 16 | 0.001 ($\downarrow$) | 5 | Batch | 40 |  | 128 | 1* | 0.0047 |
|  | 16 | 0.001 ($\downarrow$) | 5 | Batch | 40 |  | 128 | 3* | 0.0049 |
|  | 16 | 0.001 ($\downarrow$) | 5 | Batch | 40 |  | 128 | 6* | 0.00507 |
|  | 16 | 0.001 ($\downarrow$) | 5 | Batch | 40 |  | 128 | 9* | 0.00601 |
