# Supplementary material for: DeepChIA-PET: Accurately predicting ChIA-PET from Hi-C and ChIP-seq with deep dilated networks
Source: PLoS Comput Biol. 2023 Jul 13;19(7):e1011307. doi: 10.1371/journal.pcbi.1011307 (PMC10368233; doi:10.1371/journal.pcbi.1011307)
Supplement: S5 Table — The number 2 in Group Normalization is the number of groups we used. (DOCX) [file pcbi.1011307.s006.docx]

**S5 Table.** Results for hyperparameter tuning of residual networks at 5-kb resolution. The number 2 in Group Normalization is the number of groups we used.

| Mid | Batch size | Learning rate | Kernel size | Norm | No. of residual blocks | Dilation for residual blocks | Hidden dimension | Validation loss |
| --- | --- | --- | --- | --- | --- | --- | --- | --- |
| 1 | 16 | 0.001 ($\downarrow$) | 3 | Batch | 20 | [1,1,2,1,1,4,1,1,8,1,  1,16,1,1,32,1,1,64,1,1] | 64 | 0.00218 |
| 2 | 8 | 0.001 ($\downarrow$) | 5 | Batch | 20 |  | 64 | 0.00216 |
| 3 | 8 | 0.001 ($\downarrow$) | 7 | Batch | 20 |  | 64 | 0.00218 |
| 4 | 8 | 0.001 ($\downarrow$) | 5 | Batch | 22 | [1,1,2,1,1,4,1,1,8,1,  1,16,1,1,32,1,1,64,1,1,1,1] | 64 | 0.00217 |
| **5** | **8** | **0.001 (**$\boldsymbol{\downarrow}$**)** | **3** | **Batch** | **22** |  | **64** | **0.00215** |
| 6 | 16 | 0.001 ($\downarrow$) | 3 | Group（2） | 20 | [1,1,2,1,1,4,1,1,8,1,  1,16,1,1,32,1,1,64,1,1] | 64 | 0.0022 |
