# Supplementary material for: DeepChIA-PET: Accurately predicting ChIA-PET from Hi-C and ChIP-seq with deep dilated networks
Source: PLoS Comput Biol. 2023 Jul 13;19(7):e1011307. doi: 10.1371/journal.pcbi.1011307 (PMC10368233; doi:10.1371/journal.pcbi.1011307)
Supplement: S6 Table — The training data are extracted from chromosome 3 up to X and validation data from chromosome 2 for predicting CTCF ChIA-PET in GM12878. (DOCX) [file pcbi.1011307.s007.docx]

**S6 Table.** Results for hyperparameter tuning of axial attention networks at 10-kb resolution. The training data are extracted from chromosome 3 up to X and validation data from chromosome 2 for predicting CTCF ChIA-PET in GM12878.

| Mid | No. of heads | No. of blocks | Validation loss |
| --- | --- | --- | --- |
| 1 | 1 | 2 | 0.00472 |
| 2 | 1 | 4 | 0.00482 |
| 3 | 1 | 6 | 0.00479 |
| 4 | 2 | 4 | 0.00468 |
| **5** | **4** | **2** | **0.00463** |
