# Supplementary figures and images for: DeepChIA-PET: Accurately predicting ChIA-PET from Hi-C and ChIP-seq with deep dilated networks
Source: PLoS Comput Biol. 2023 Jul 13;19(7):e1011307. doi: 10.1371/journal.pcbi.1011307 (PMC10368233; doi:10.1371/journal.pcbi.1011307)

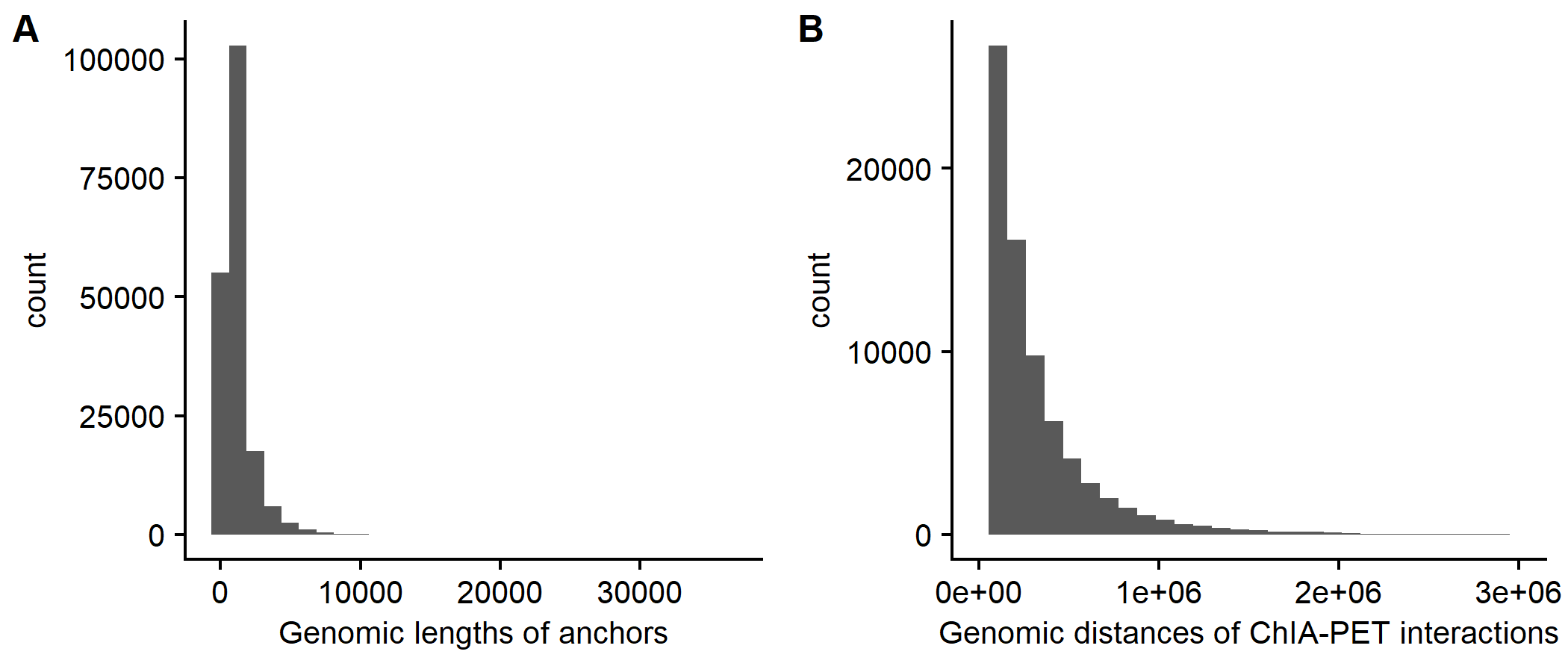

Supplement: S1 Fig — For CTCF ChIA-PET interactions in GM12878, we find (A) almost all anchors’ genomic lengths are less than 10 kb, and (B) almost all ChIA-PET interactions are within 2 Mb. (TIFF) [file pcbi.1011307.s008.tiff]

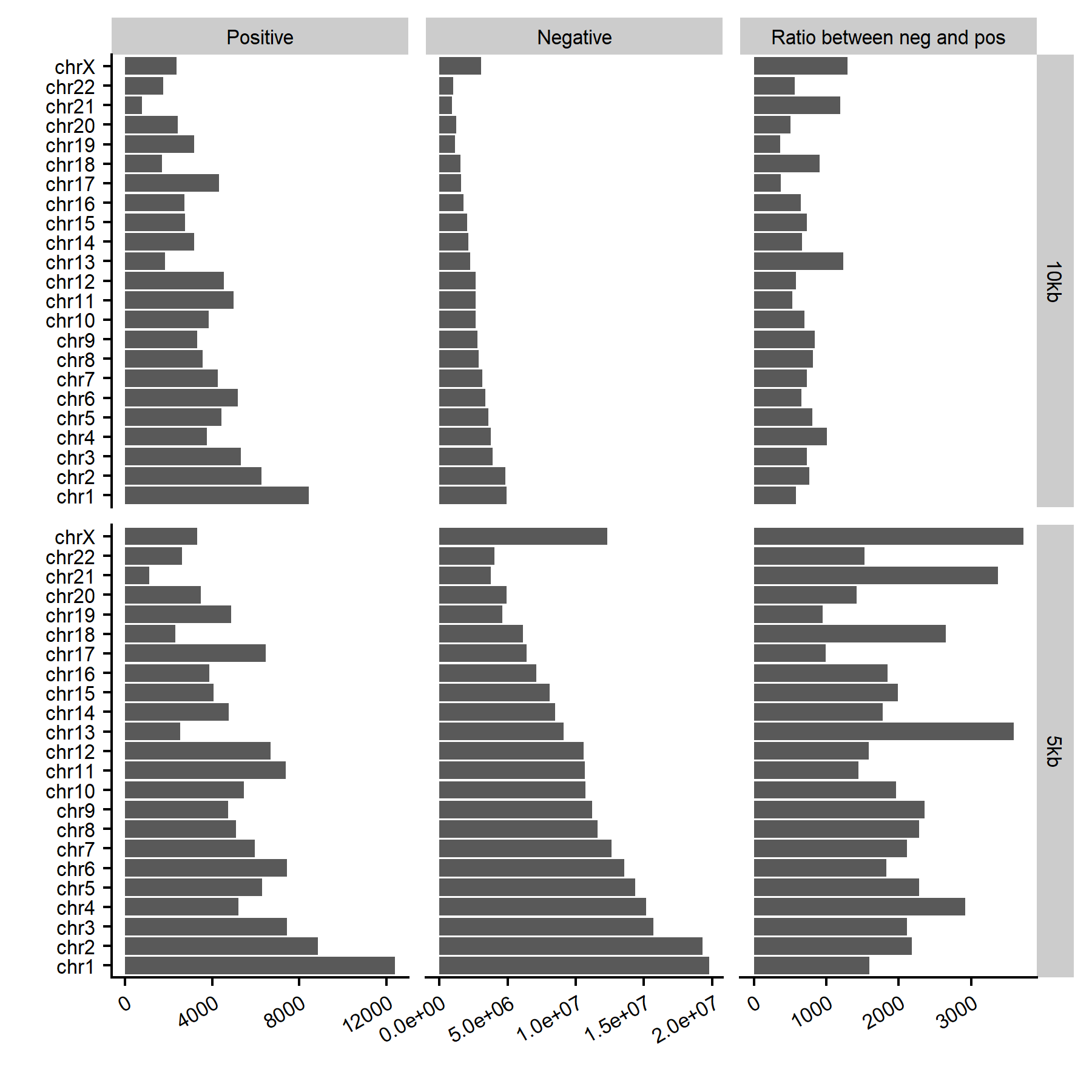

Supplement: S2 Fig — The range of genomic distances we cared about is from 2 to 200 bins for 10 kb and from 2 to 400 for 5 kb, that is, 20 kb to 2 Mb. The ChIA-PET data are from CTCF of GM12878. (TIFF) [file pcbi.1011307.s009.tiff]

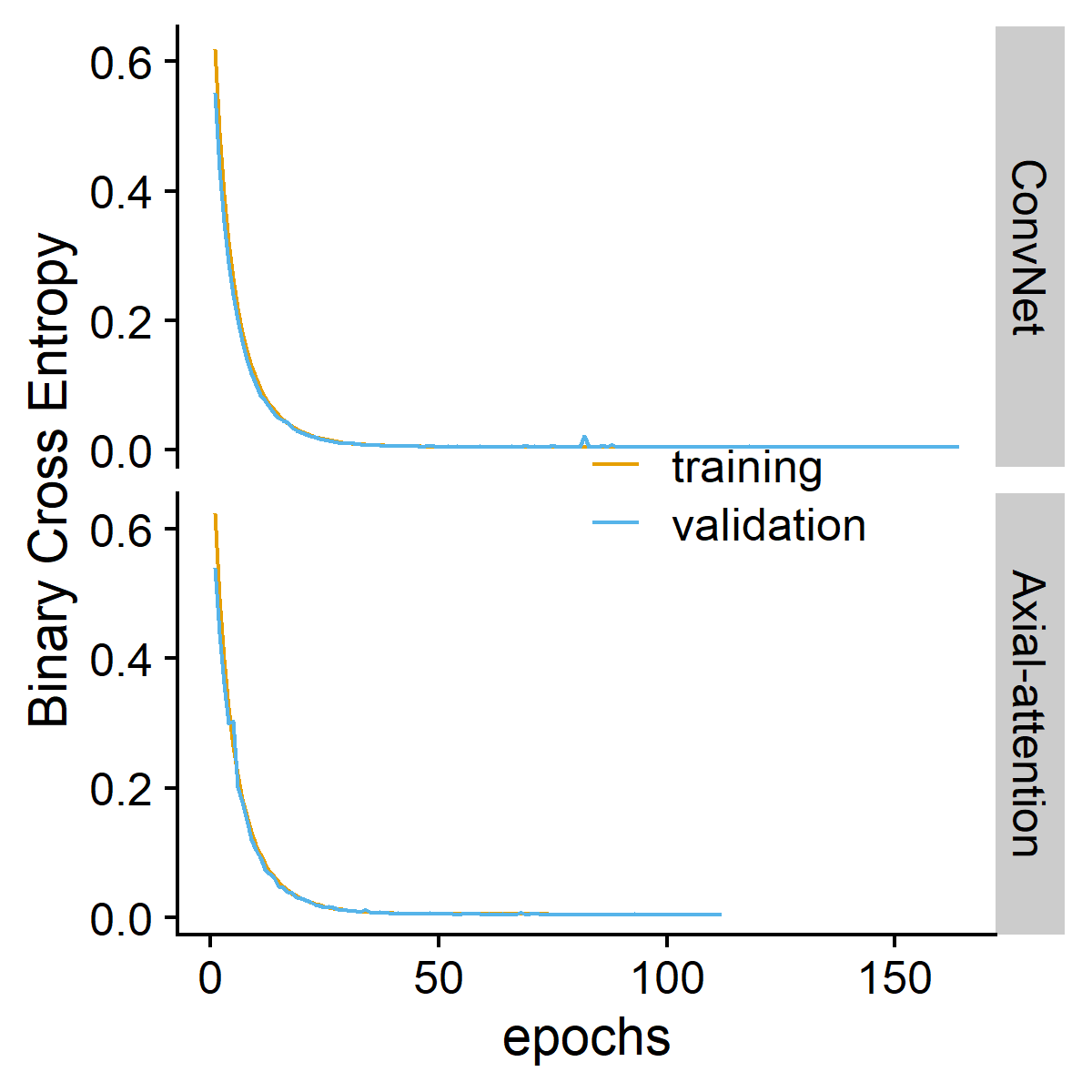

Supplement: S3 Fig — The learned model was trained for blindly testing on chromosome 1. The validation data were extracted from chromosome 2. The training data were generated from the rest of the chromosome from 3 to X. (TIFF) [file pcbi.1011307.s010.tiff]

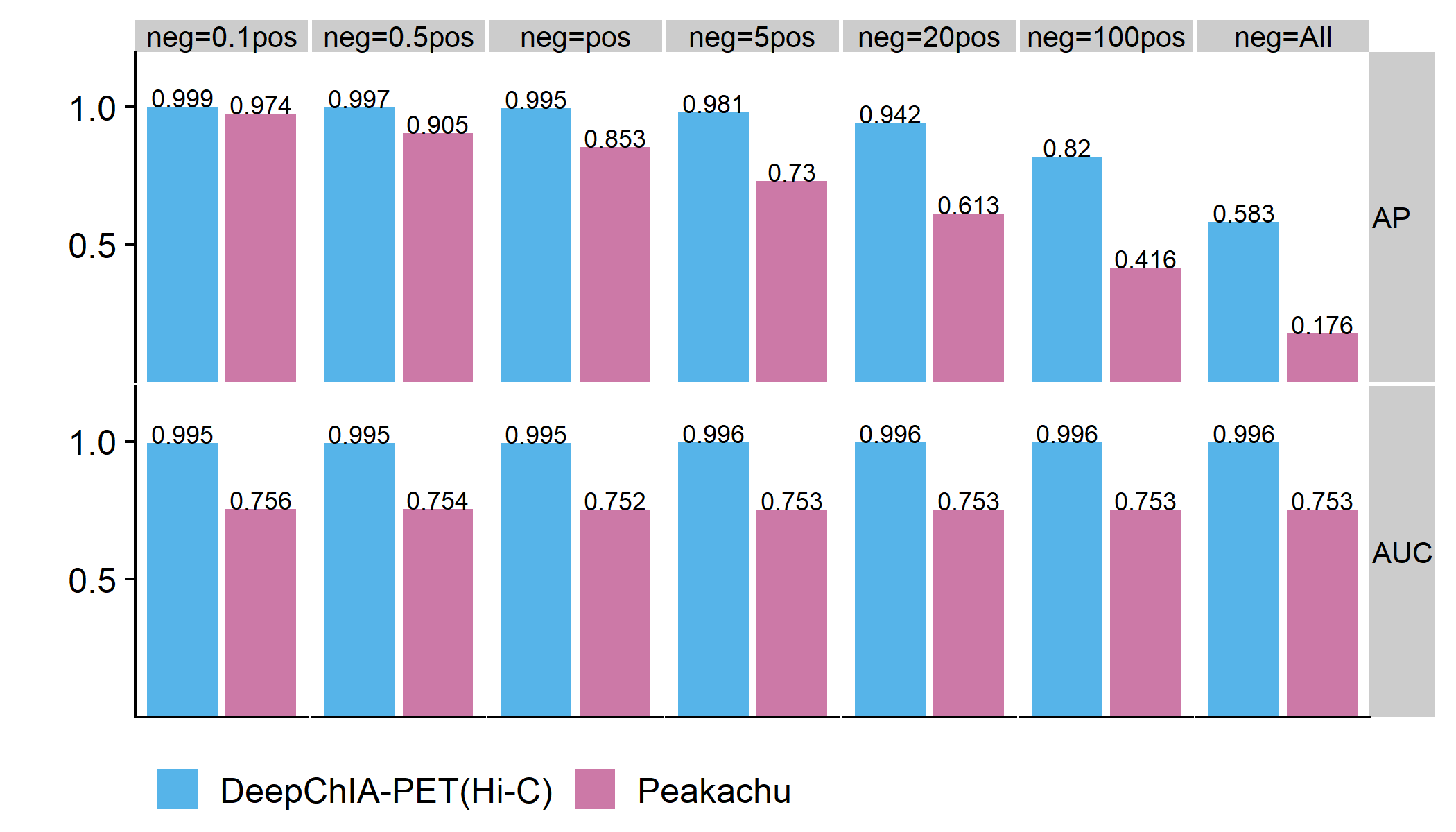

Supplement: S4 Fig — DeepChIA-PET(Hi-C) outperforms Peakachu for blind testing CTCF ChIA-PET on chromosome 1 in GM12878 in terms of AP and AUC at 10-kb resolution. (TIFF) [file pcbi.1011307.s011.tiff]

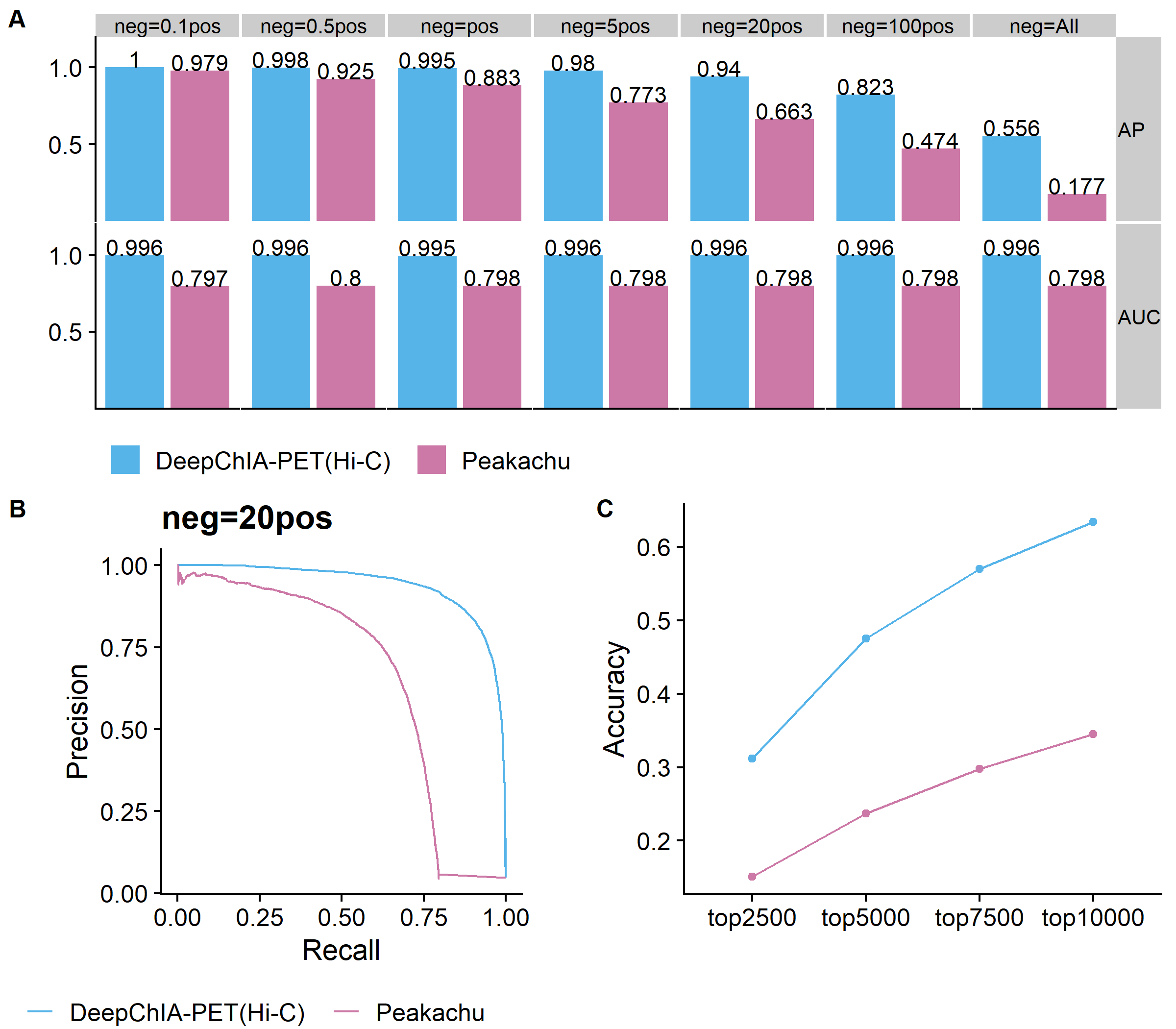

Supplement: S5 Fig — (A) The AP and AUC results for different number of negative pixels used for evaluating. (B) Precision-recall curve for the number of negative pixels equal to 20 times the number of positive pixels. (C) The top-N accuracy where N equals four different values. (TIFF) [file pcbi.1011307.s012.tiff]

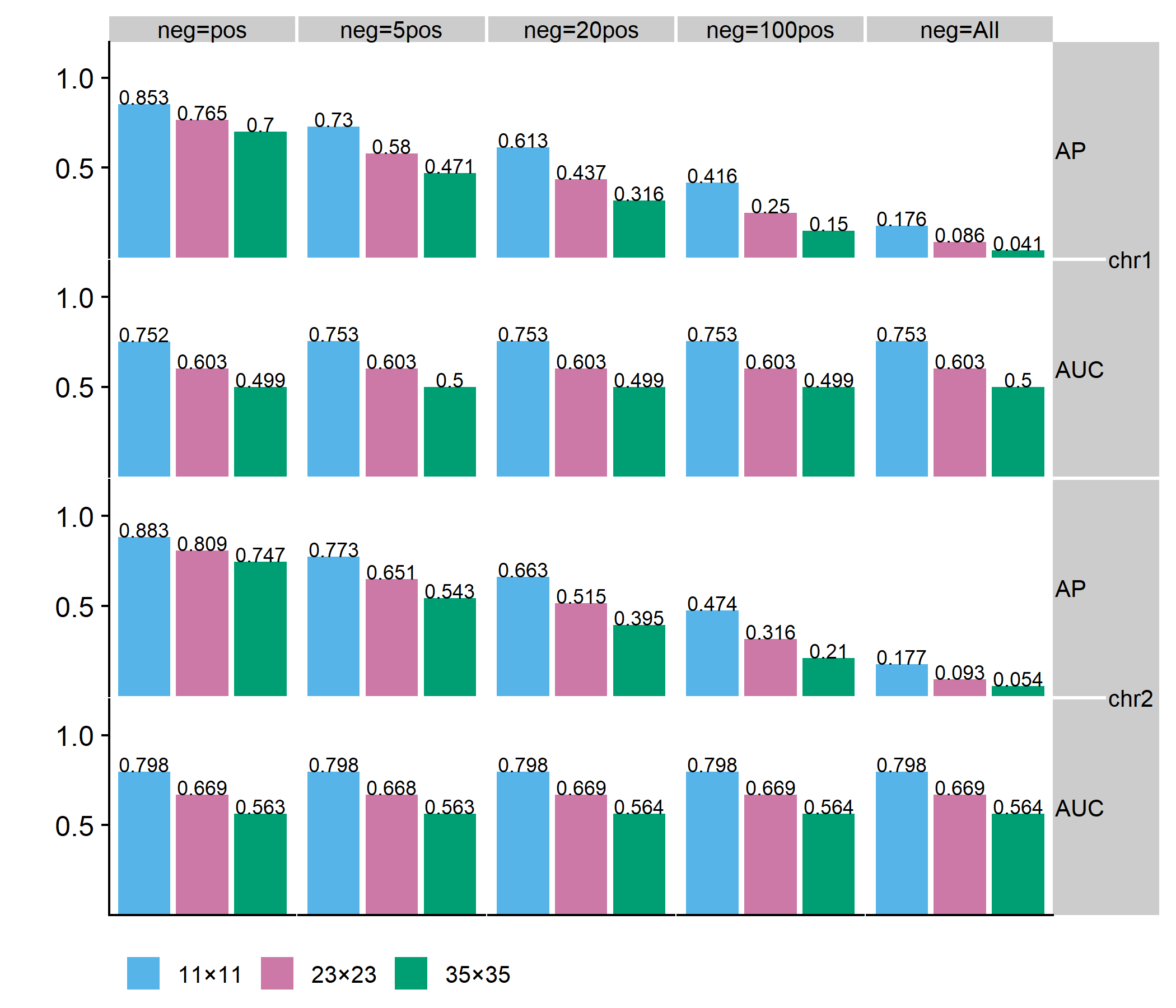

Supplement: S6 Fig — The AP and AUC results for the three window sizes are from blindly testing CTCF ChIA-PET on chromosomes 1 and 2 in GM12878 at 10-kb resolution. (TIFF) [file pcbi.1011307.s013.tiff]

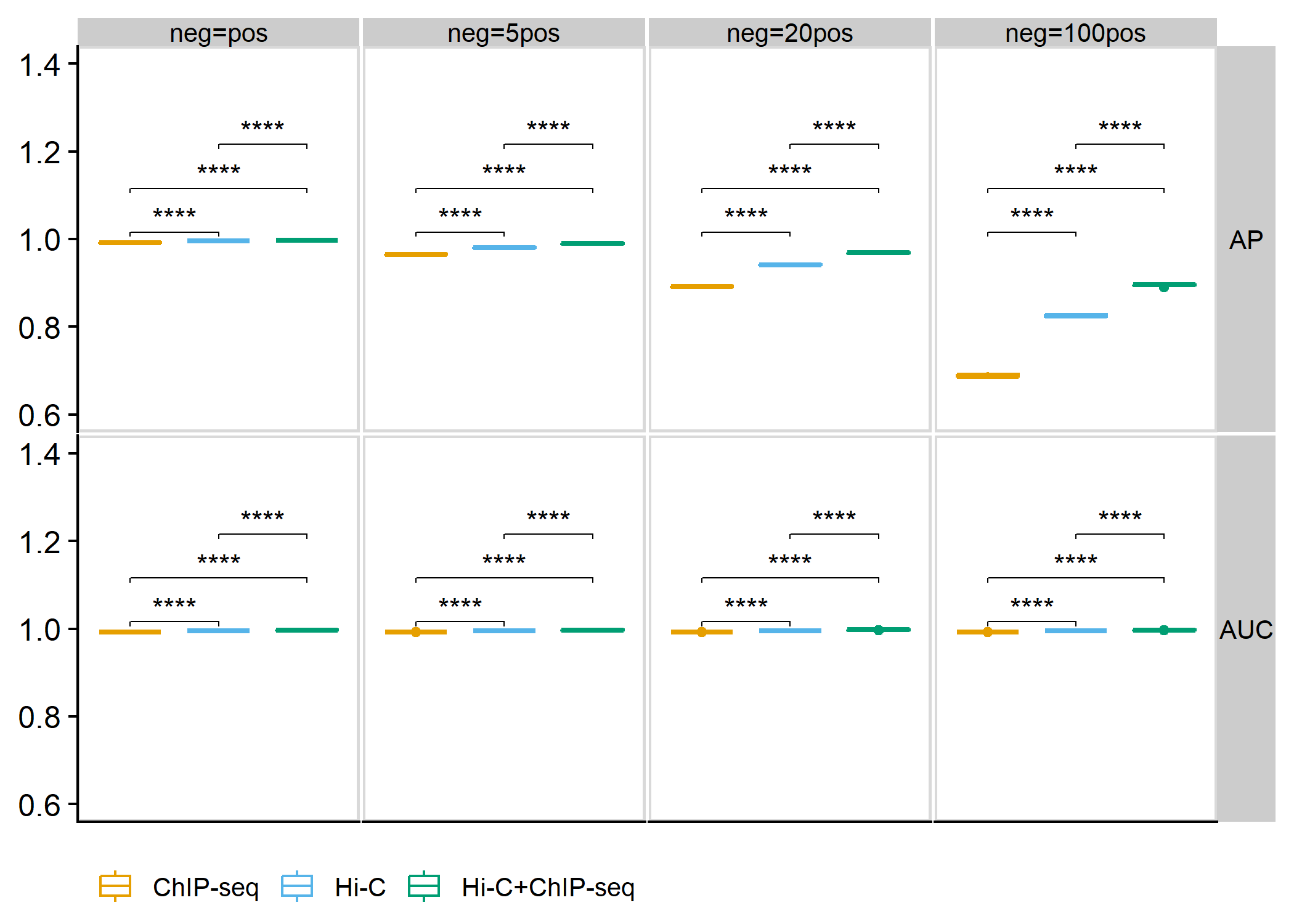

Supplement: S7 Fig — Each negative set is randomly generated 10 times. ****: p-value < = 0.0001. The evaluation for DeepChIA-PET with only Hi-C, only ChIP-seq, and both as input is conducted on chromosome 2 for CTCF ChIA-PET in GM12878 at 10-kb resolution. (TIFF) [file pcbi.1011307.s014.tiff]

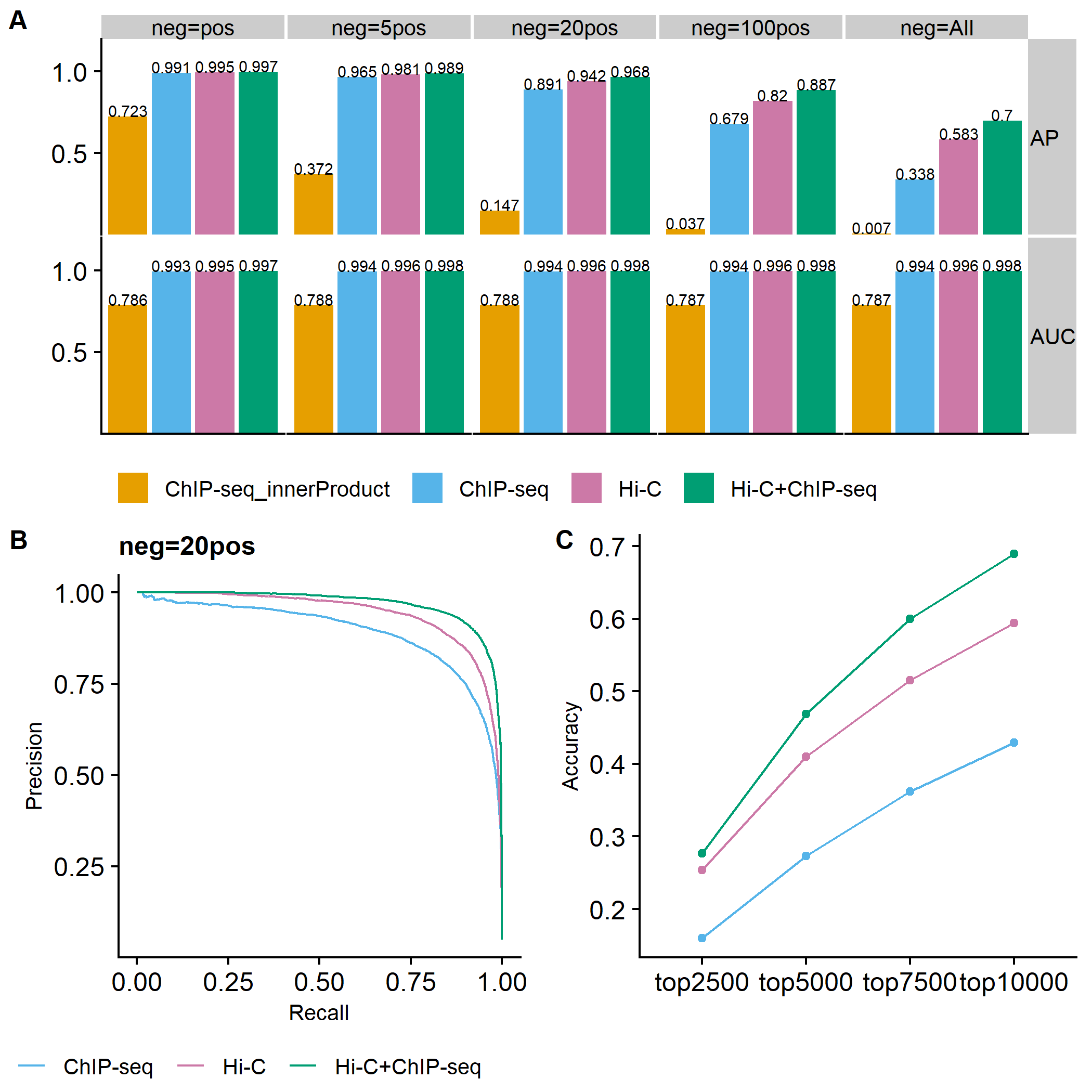

Supplement: S8 Fig — The evaluation for DeepChIA-PET with only Hi-C, only ChIP-seq, and both as input is conducted on chromosome 1 for CTCF ChIA-PET in GM12878 at 10-kb resolution. (A) The AP and AUC results for different number of negative pixels used for evaluating. The inner product of ChIP-seq is provided as a baseline. (B) Precision-recall curve for the number of negative pixels equal to 20 times number of positive pixels. (C) The top-N accuracy where N equals four different values. (TIFF) [file pcbi.1011307.s015.tiff]

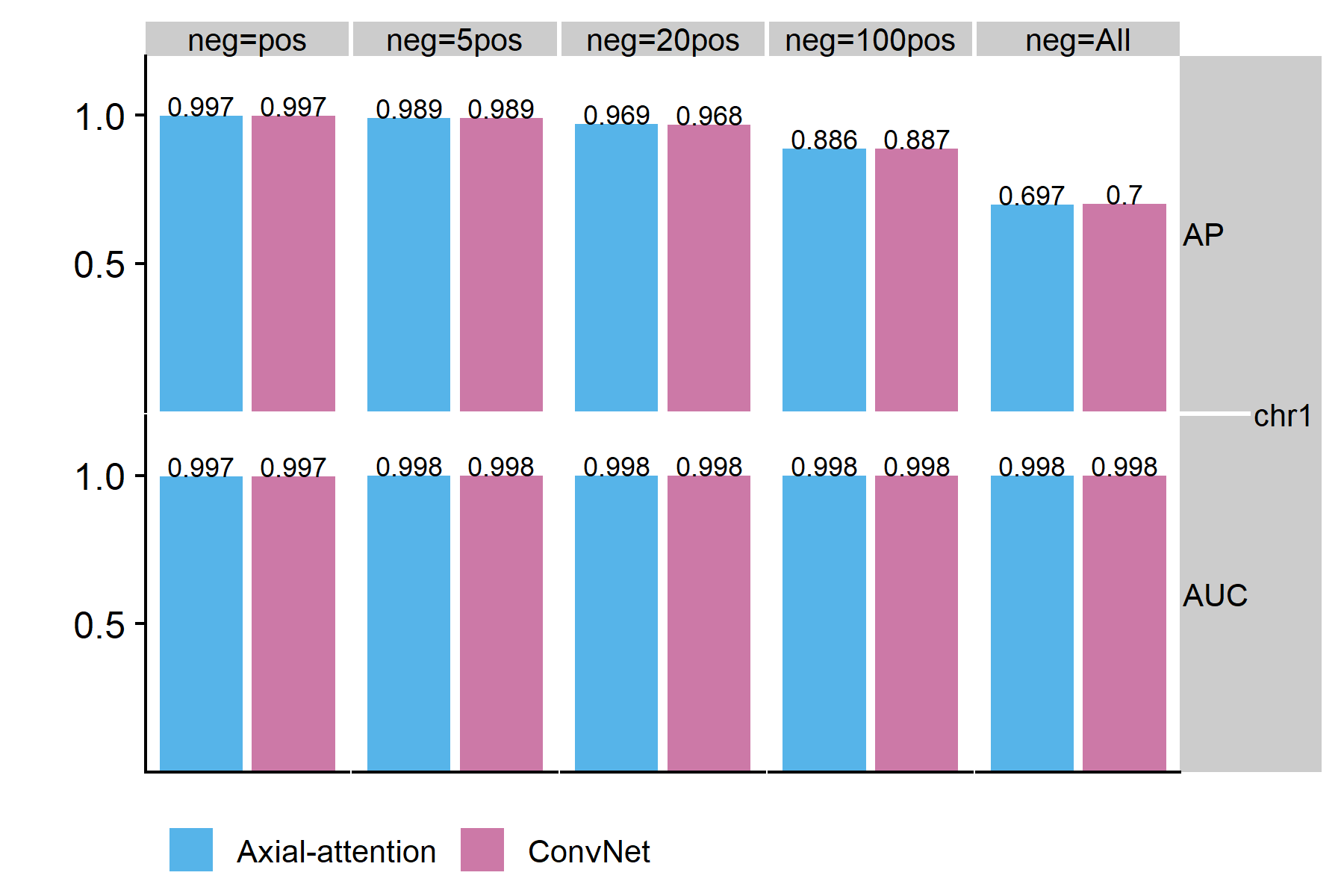

Supplement: S9 Fig — The AP and AUC values are calculated for testing CTCF ChIA-PET on chromosome 1 in GM12878 at 10-kb resolution. (TIFF) [file pcbi.1011307.s016.tiff]

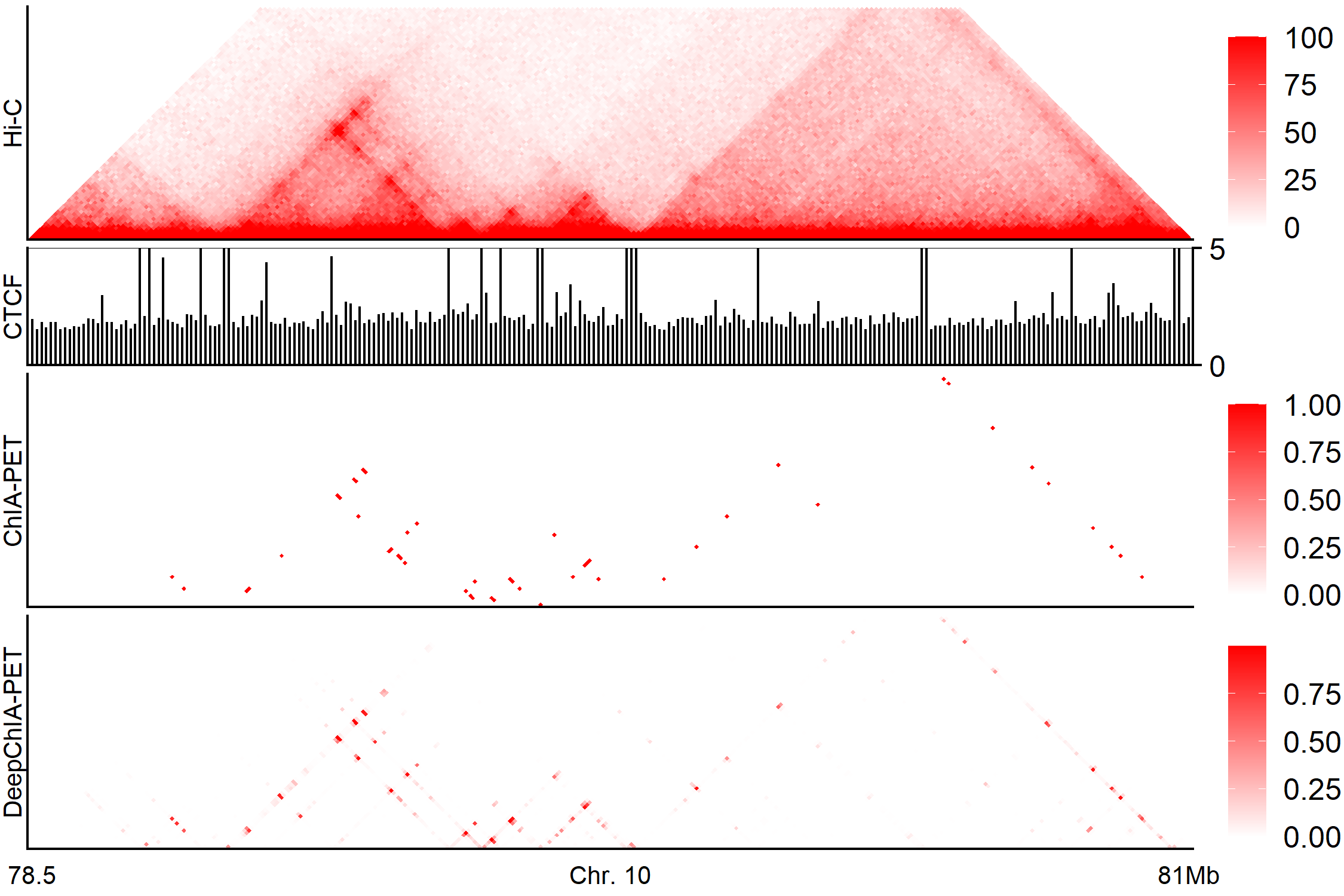

Supplement: S10 Fig — From top to bottom: KR-normalized Hi-C, CTCF, ground-truth ChIA-PET, and our predicted ChIA-PET. (TIFF) [file pcbi.1011307.s017.tiff]

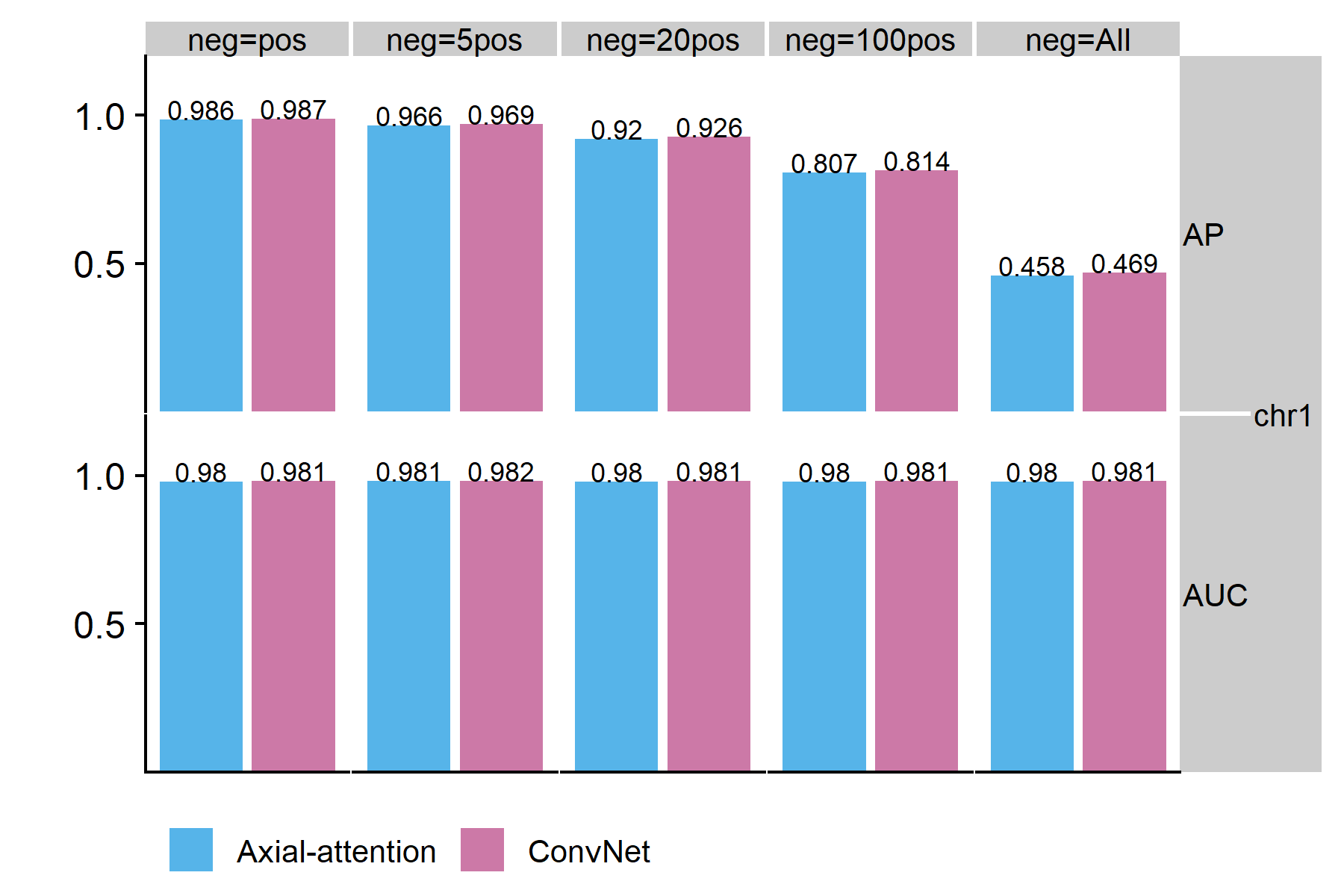

Supplement: S11 Fig — The AP and AUC values are calculated for testing CTCF ChIA-PET on chromosome 1 in HeLa at 10-kb resolution. (TIFF) [file pcbi.1011307.s018.tiff]

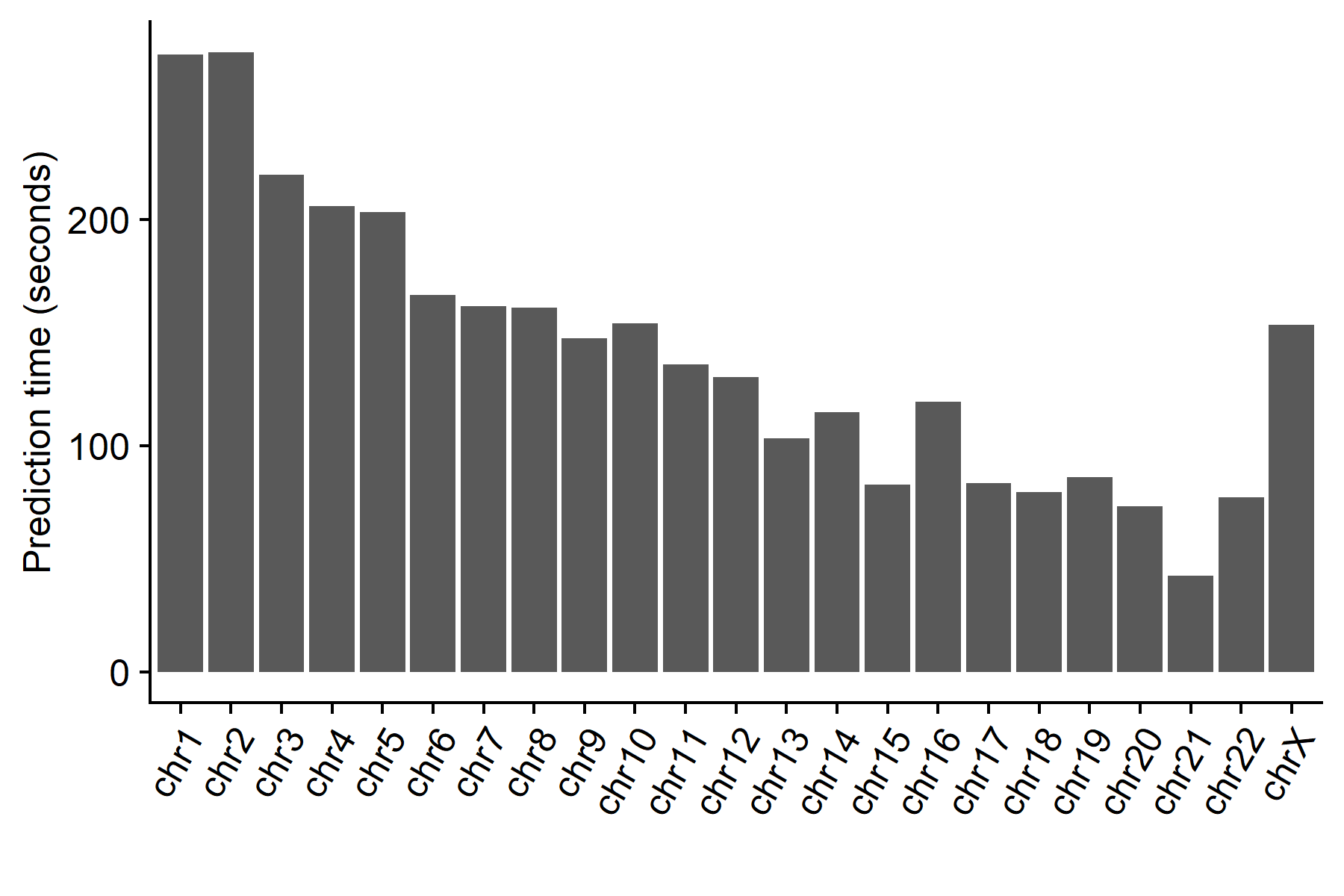

Supplement: S12 Fig — For predicting each chromosome, we set the batch size to 2 and used four NVIDIA A100 GPUs (each equipped with 40GB memory) in parallel. (TIFF) [file pcbi.1011307.s019.tiff]

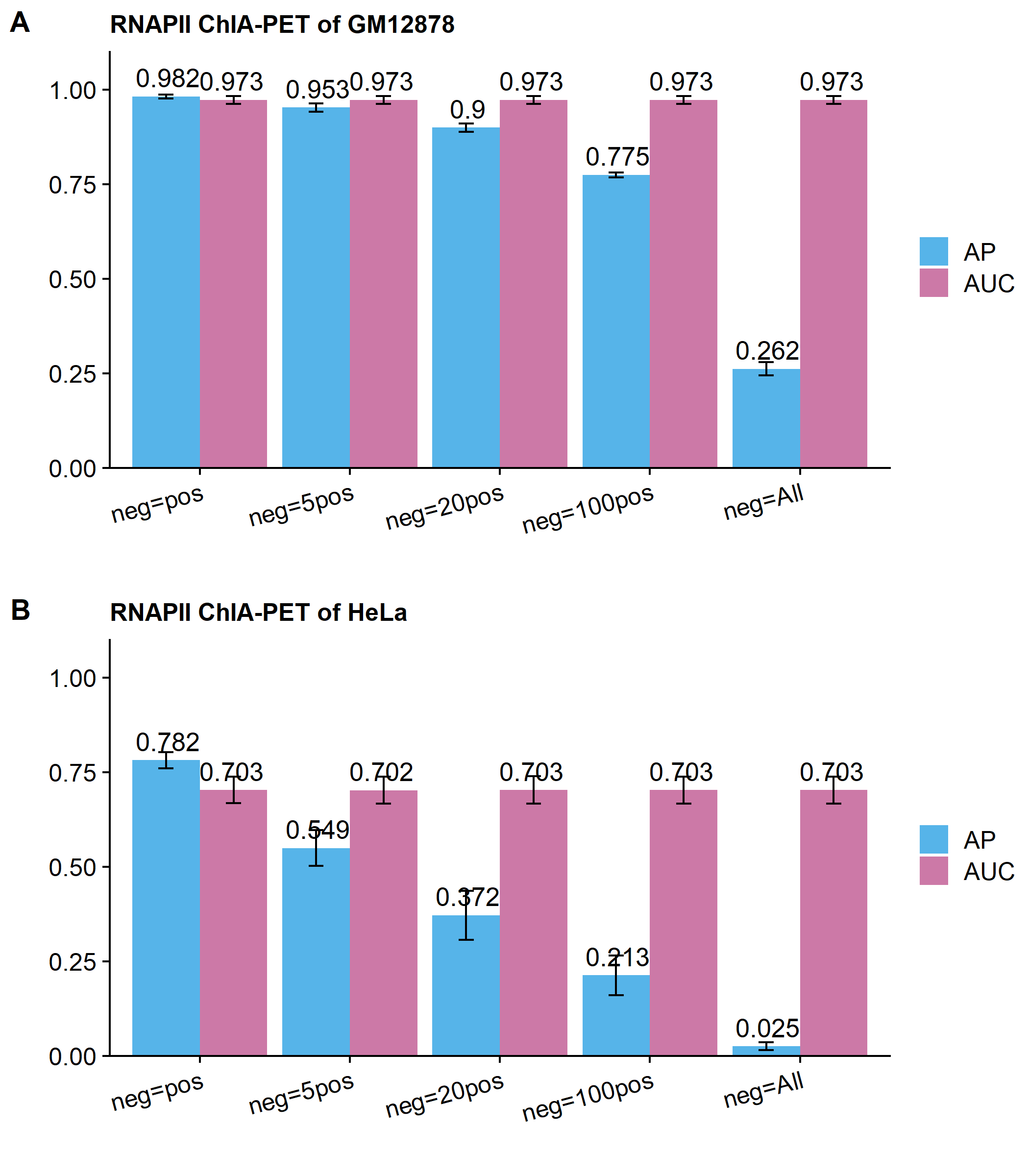

Supplement: S13 Fig — Error bars show standard deviation (SD) with n = 5. (TIFF) [file pcbi.1011307.s020.tiff]

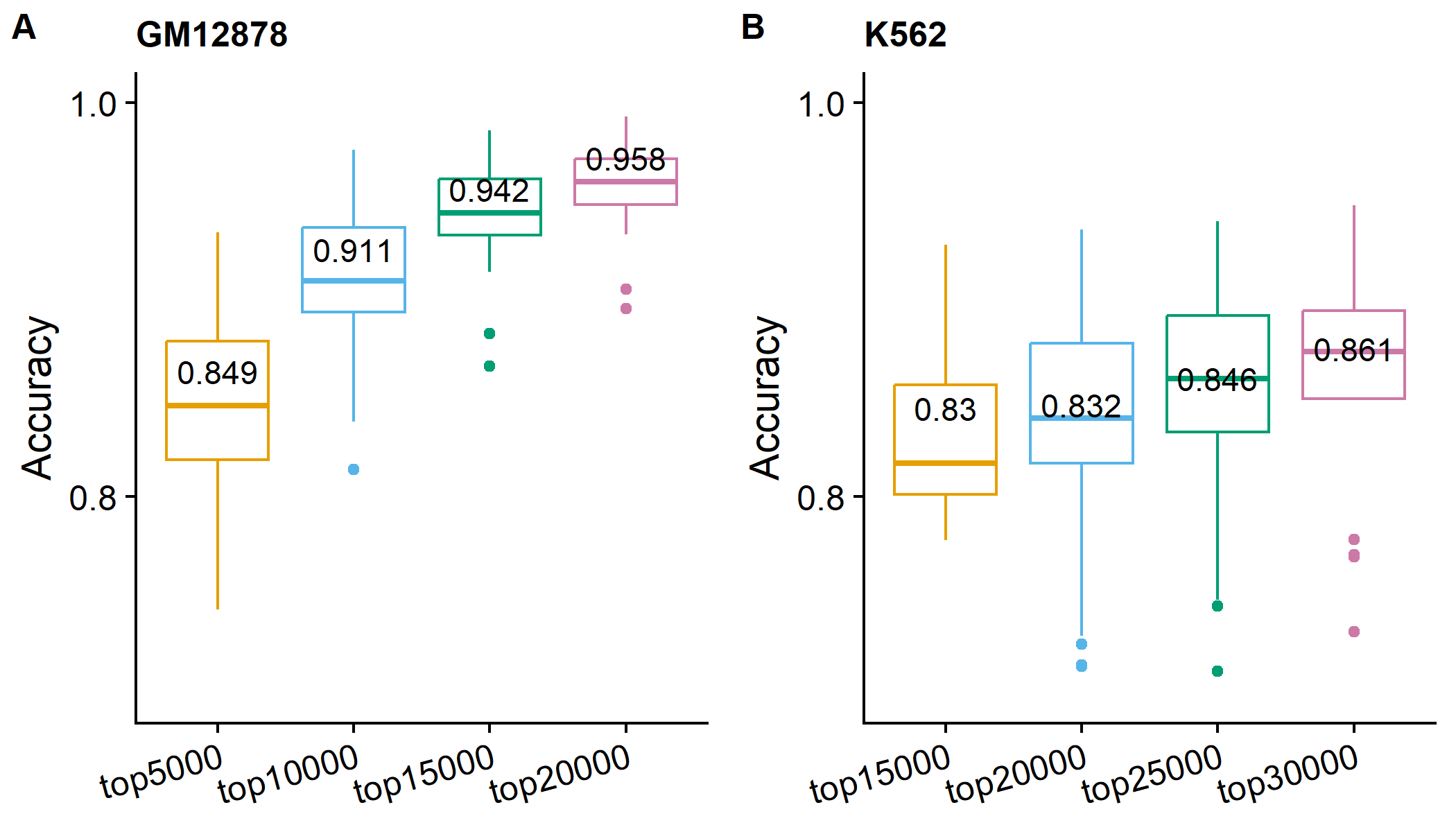

Supplement: S14 Fig — Both (A) and (B) were generated on all testing chromosomes (mean values were added above each boxplot). Note: since the Hi-C matrix for chromosome 9 in K562 is empty, the evaluation results shown in (B) do not include the accuracy score for chromosome 9. (TIFF) [file pcbi.1011307.s021.tiff]

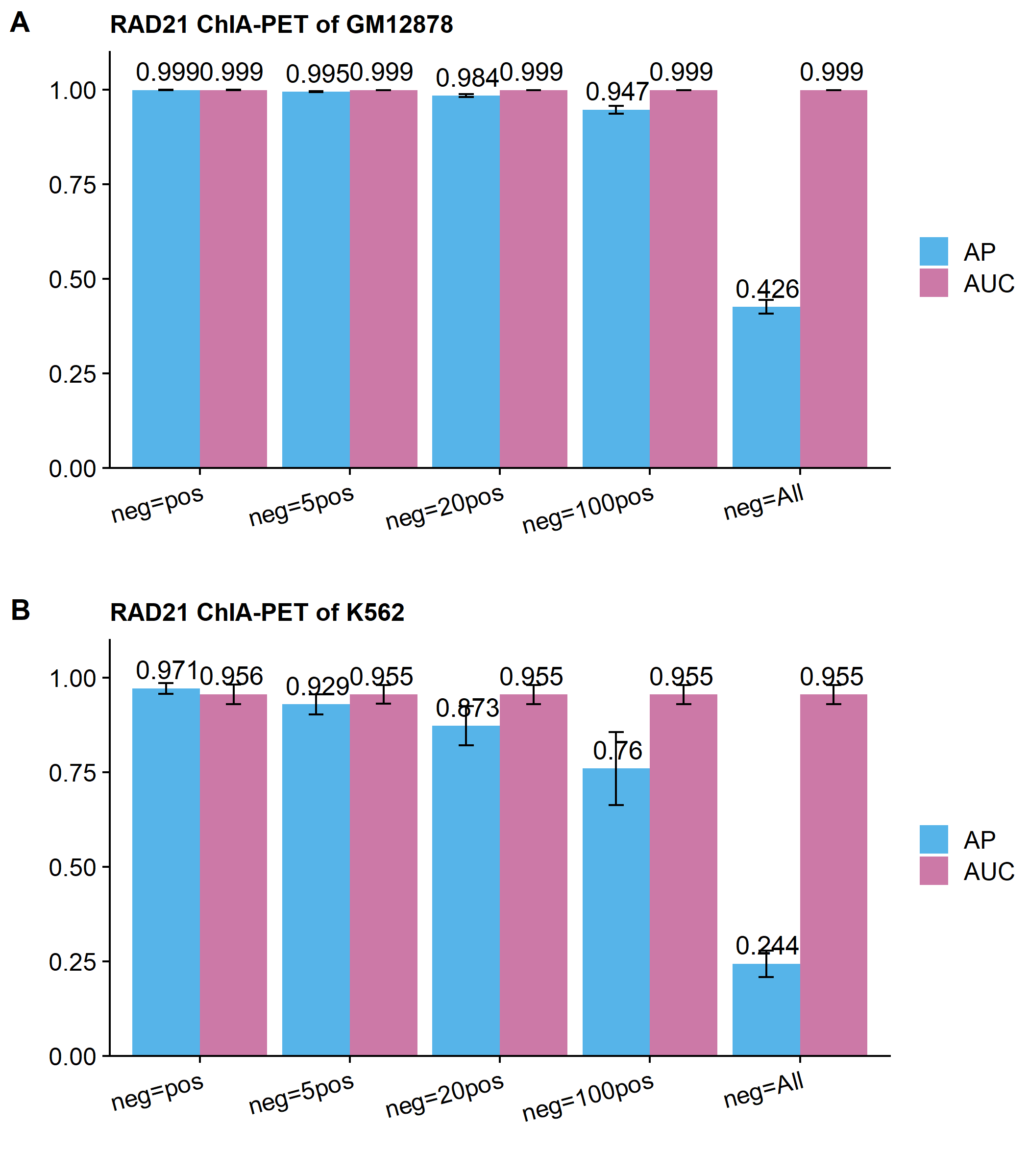

Supplement: S15 Fig — Error bars show standard deviation (SD) with n = 5. (TIFF) [file pcbi.1011307.s022.tiff]

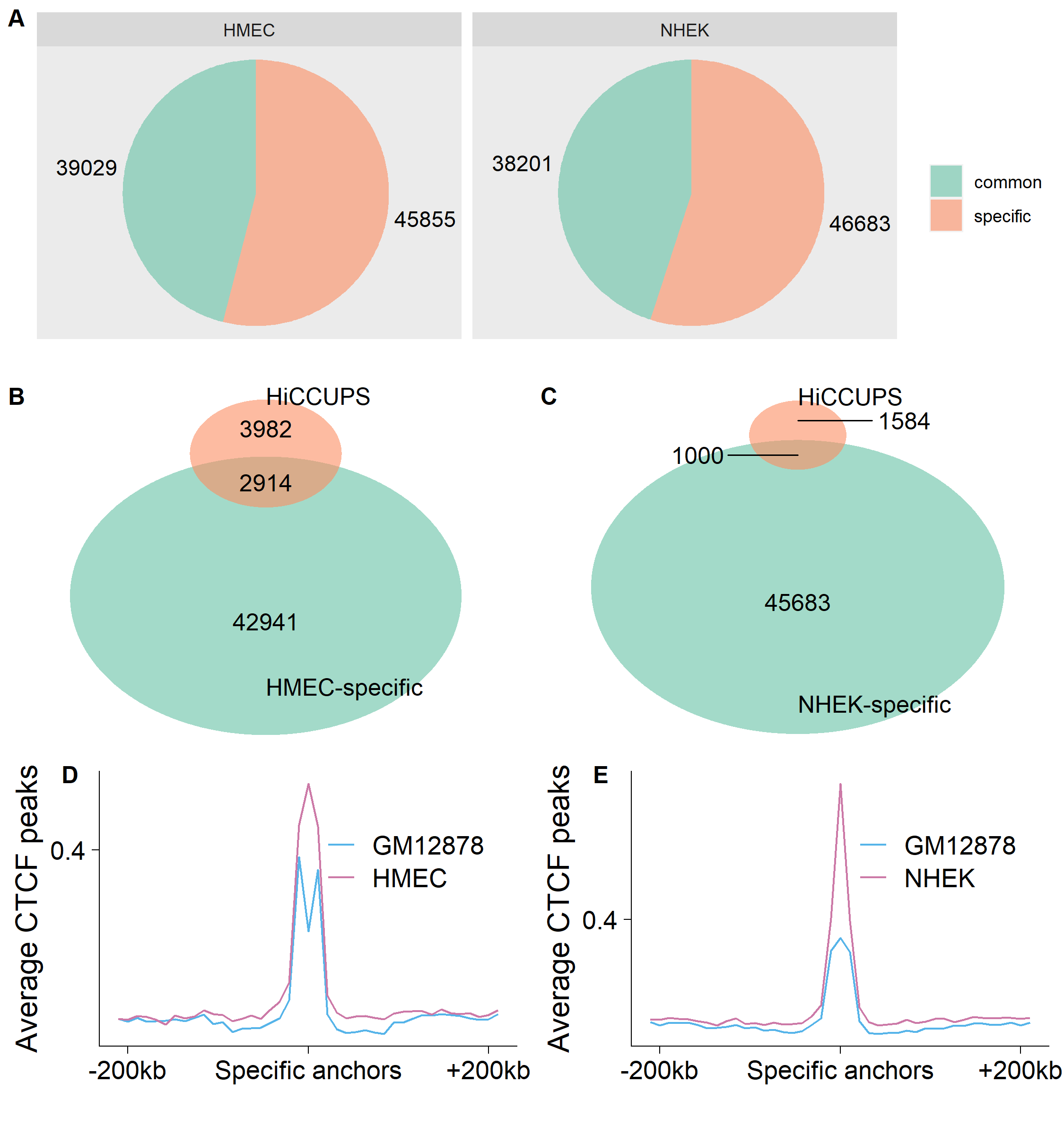

Supplement: S16 Fig — (A) compared with CTCF ChIA-PET of GM12878, the number of common and specific loops for HMEC and NHEK. For each chromosome, the top-N loops are considered. Here N indicates the number of loops in GM12878. Genome-wide overlaps between Hi-C peaks called by HiCCUPS and HMEC-specific CTCF ChIA-PET interactions (B), and between Hi-C peaks called by HiCCUPS and NHEK-specific CTCF ChIA-PET interactions (C). When counting the accordant pixels between ChIA-PET and Hi-C peaks, we allow ± 1 bin mismatch. The specific anchors from HMEC (D) and NHEK (E) are enriched for CTCF peaks compared with GM12878. (TIFF) [file pcbi.1011307.s023.tiff]
